# Ultra‐Thin Lead Sulfide Capping for Stabilizing Tin Halide Perovskite Solar Cells

**DOI:** 10.1002/smll.202506340

**Published:** 2025-08-23

**Authors:** Pok Fung Chan, Yongmin Luo, Haiyang Cheng, Xuezhou Wang, Ni Zhao, Jiaying Wu, Chun‐Jen Su, Jhih‐Min Lin, Hengkai Zhang, Xinhui Lu

**Affiliations:** ^1^ Department of Physics The Chinese University of Hong Kong New Territories Hong Kong SAR 999077 China; ^2^ Adv. Mater. Thrust, Function Hub Hong Kong University of Science and Technology (Guangzhou) Guangzhou 511458 China; ^3^ Department of Electronic Engineering The Chinese University of Hong Kong New Territories Hong Kong SAR 999077 China; ^4^ National Synchrotron Radiation Research Center Hsinchu Science Park Hsinchu 30076 Taiwan

**Keywords:** efficient, inorganic layer, lead‐free perovskite, stable

## Abstract

Addressing the inherent instability of tin‐based perovskite solar cells (PSCs), a facile approach is introduced to simultaneously enhance their stability and performance. This study demonstrates the implementation of an ultra‐thin (≈3 nm) dual‐functional lead sulfide (PbS) capping layer, formed via a sequential deposition. This PbS layer serves a critical dual role: first, as a robust protective barrier against oxidation and moisture, effectively mitigating the degradation of the underlying tin‐based perovskite; and second, by improving interfacial electronic properties to facilitate efficient charge extraction. Specifically, the PbS layer not only provides conformal coverage and minimizes non‐radiative recombination but also acts as an n‐type interfacial layer, promoting electron transport. This synergistic effect results in a significant boost in power conversion efficiency (PCE) from 12.0% to 14.4% with an open‐circuit voltage of 0.845 V, and exceptional long‐term stability with negligible PCE decay after 1600 h in a nitrogen environment. Operando grazing‐incidence wide‐angle X‐ray scattering (GIWAXS) measurements corroborate enhanced structural stability under operating conditions. These findings establish the PbS capping layer as a practical, facile, and highly effective strategy for advancing stable and high‐performance tin‐based PSCs, paving the way for their viable application in sustainable photovoltaics.

## Graphical abstract

An ultra‐thin (≈3 nm) dual‐functional lead sulfide (PbS) capping layer, formed via a facile sequential spin‐coating and annealing process, is designed as a protective layer for FASnI_3_. This PbS layer serves a critical dual role: first, as a robust protective barrier against oxidation and moisture; and second, by improving interfacial electronic properties to facilitate efficient charge extraction. This synergistic effect results in a significant boost in power conversion efficiency (PCE) from 12.0% to 14.4%.

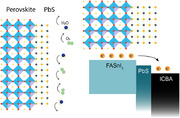

## 1 Introduction

Lead‐based perovskite solar cells (PSCs) have achieved remarkable advancements over the past 15 years, reaching a competitive record power conversion efficiency (PCE) of 27.0%.^[^ ^1^ ^]^ However, the inherent toxicity of lead and the associated environmental hazards pose significant challenges to the scalability and commercialization of lead‐based PSCs.^[^ ^2^ ^]^ In this context, tin‐based perovskites have emerged as a viable alternative, owing to the similar ionic radii and electronic configurations of Sn^2+^ and Pb^2+^, along with favorable optoelectronic properties, such as optimal band gaps,^[^ ^3^ ^]^ enhanced carrier mobilities,^[^ ^4^ ^]^ and prolonged hot carrier relaxation.^[^ ^5^ ^]^ Despite these promising attributes, the development of tin‐based PSCs is hindered by their inherent instability, particularly their susceptibility to oxidation in ambient conditions, which limits their PCE to below 17%.^[^ ^6^, ^7^, ^8^ ^]^ The instability of tin‐based perovskites primarily results from the oxidation of tin(II) (Sn^2^⁺) to tin(IV) (Sn⁴⁺) in the presence of oxygen and moisture.^[^ ^9^ ^]^ Such oxidation disrupts the perovskite structure and generates defects that act as recombination centers, severely impairing device performance.^[^ ^4^, ^10^ ^]^ Moreover, uncontrolled crystallization during film formation often results in poor film quality, further exacerbating stability issues.^[^ ^11^ ^]^

Efforts to enhance the stability of tin‐based perovskites have largely focused on surface treatments that passivate dangling bonds with organic molecules. An ideal surface or capping layer for PSCs, especially for tin‐based systems, should meet several key requirements: 1) it must form a conformal coverage to isolate the perovskite from the ambient environment, 2) it should provide strong charge selectivity for efficient charge extraction, and 3) it should form a defect‐free interface with the perovskite film to minimize non‐radiative recombination.

One widely explored approach involves utilizing bulky organic ligands, such as phenylethylammonium (PEA) or butylammonium (BA), to create a protective 2D perovskite layer on the 3D tin‐based perovskite surface.^[^ ^12^ ^]^ However, this approach has shown limited improvement in the quality of the underlying tin‐based perovskite film,^[^ ^13^ ^]^ and the 2D layer can introduce additional energy barriers that hinder charge extraction.^[^ ^14^ ^]^ Alternatively, additives and surface passivation agents have been employed to suppress oxidation and enhance film quality. For instance, reducing agents such as sulfonic acid or hydrazine derivatives have been incorporated into the perovskite precursor to mitigate Sn^2^⁺ oxidation.^[^ ^15^, ^16^ ^]^ Surface passivation with organic molecules, such as hydrazide^[^ ^17^ ^]^ or thiourea derivatives,^[^ ^18^ ^]^ has also been applied to address surface defects and reduce non‐radiative recombination. Despite these improvements, these organic molecules do not form a compact layer to isolate air and moisture from the perovskite films, limiting their long‐term resistance to oxidation.

In contrast, inorganic layers, such as lead sulfate,^[^ ^19^ ^]^ lead sulfide (PbS)^[^ ^20^, ^21^, ^22^ ^]^ and tin selenide,^[^ ^23^ ^]^ have been reported to effectively prevent both pure lead and tin‐lead mixed perovskites from direct exposure to the ambient environment, thereby significantly improving the overall stability. However, this strategy has yet to be reported for pure tin‐based perovskite systems, mainly due to the vulnerability of their surface to common solvent compositions.^[^ ^24^ ^]^

In this work, we present a straightforward method to grow an ultra‐thin (≈3 nm) conformal layer of PbS through an in situ reaction on the tin‐based perovskite film. Specifically, the PbS layer is formed by sequentially depositing a lead salt, Pb(PyA)_2_, followed by a sulfur source, (TMS)_2_S, directly onto the perovskite surface. Upon mild thermal annealing, these precursors react locally on the substrate to generate a continuous PbS layer in place. Since PbS is insoluble in water and chemically stable in ambient air, it acts as a robust barrier against oxygen and moisture, significantly retarding the oxidation of Sn^2+^. The electronic properties of the PbS layer were carefully tuned by adjusting the ratio between lead(II) pyridinecarboxylic acid salt (Pb(PyA)_2_) and bis(trimethylsilyl)sulfide ((TMS)_2_S). Kelvin probe force microscopy (KPFM) results indicate that the perovskite surface exhibits an upshifted Fermi level in the Pb‐rich condition, forming a more n‐type interface to facilitate the charge extraction to the electron transport layer (ETL). Furthermore, the presence of the PbS layer reduces intra‐grain potential inhomogeneity, preventing local charge accumulation. Notably, when being exposed to ambient conditions, the PbS layer significantly slows the deepening of the Fermi level, in other words, reduces the oxidation‐induced p‐doping, demonstrating improved oxidation resistance. Additionally, the interaction between formamidinium tin iodide (FASnI_3_) and PbS mitigates surface defects by suppressing dangling bonds at the interface. As a result, our approach leads to a considerable increase in the PCE, rising from 12.0% to 14.4%, accompanied by a significant improvement in open‐circuit voltage (*V*_OC_) from 0.727 to 0.845 V. This method offers a practical solution for enhancing the viability of tin‐based perovskite solar cells, paving the way for more sustainable photovoltaic technologies.

## 2 Results and Discussion

### 2.1 The PbS Capping Layer Fabrication and Composition Characterization

**Figure 1:**
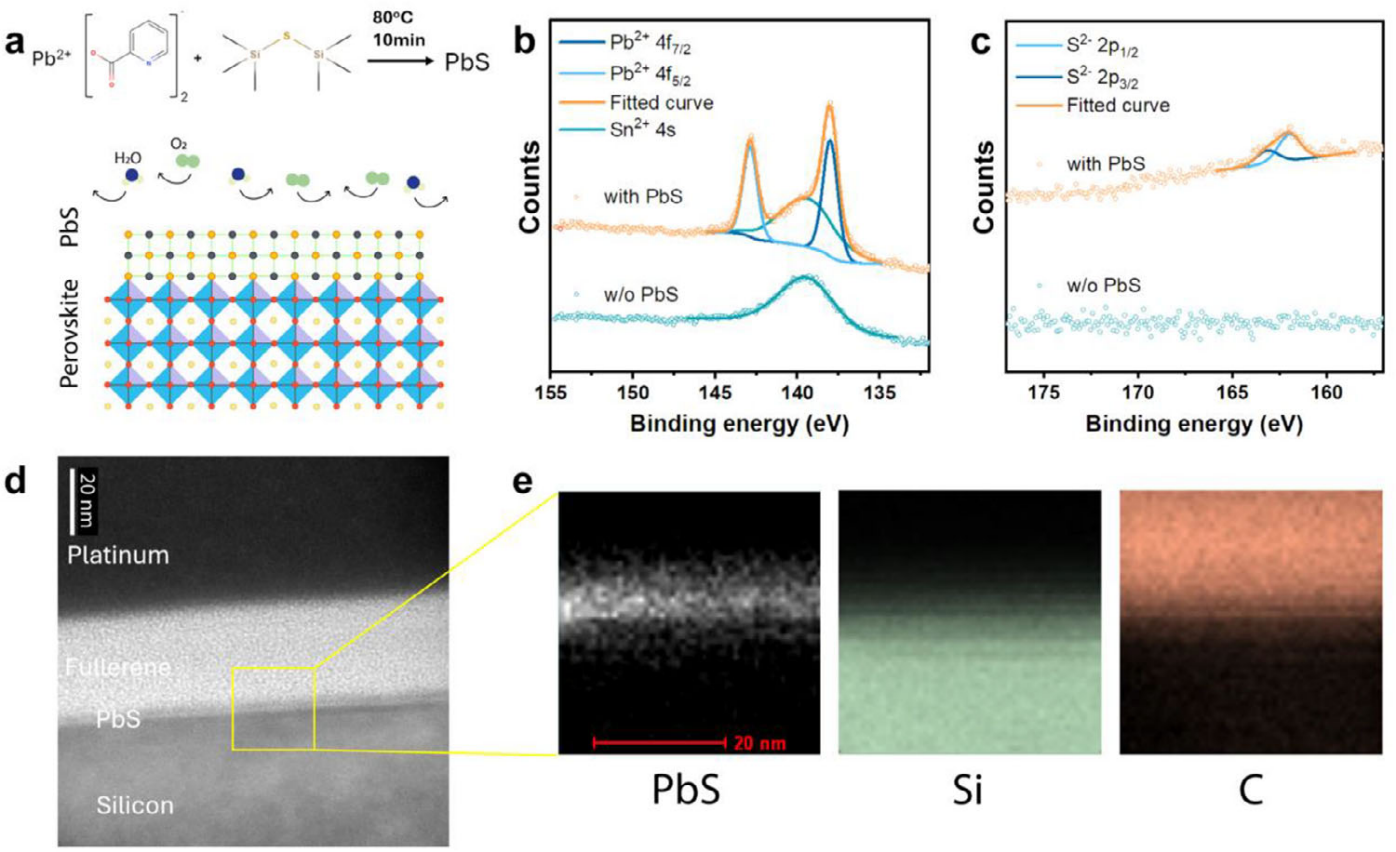
a) Schematics of the in situ synthesis of the ultra‐thin PbS layer on a FASnI_3_ film. b) Pb 4f and c) S 2p XPS spectra of the film with and the film without the PbS layer. d) Cross‐sectional TEM image of a PbS layer deposited on a silicon substrate with a precursor concentration of 1 mg mL^−1^ (Pb(PyA)_2_) and 3 mg mL^−1^ ((TMS)_2_S). e) EDX spectra mapping of the interface between the fullerene and the silicon substrate.

Previous studies have shown that PbS can be synthesized via the reaction between lead(II) pyridinecarboxylic salt (Pb(PyA)_2_) and bis(trimethylsilyl)sulfide ((TMS)_2_S).^[^ ^25^ ^]^ Therefore, we prepare the precursor solutions of Pb(PyA)_2_ in hexafluoro‐2‐propanol at a concentration of 1 mg mL^−1^ and (TMS)_2_S in CB at a concentration of 3 mg mL^−1^, based on the optimal concentrations for device performance (Figure S16, Supporting Information). Differential Scanning Calorimetry (DSC) measurements of the Pb(PyA)_2_ and (TMS)_2_S mixed solutions revealed an exothermic peak at ≈70 °C, suggesting that the reaction would happen at a temperature above 70 °C (Figure S1, Supporting Information). To form an ultra‐thin layer of PbS, the Pb(PyA)_2_ and (TMS)_2_S precursor solutions were sequentially spin‐coated onto the FASnI_3_ perovskite layer, followed by annealing at 80 °C for 10 min, as illustrated in **Figure** **1**a. The detailed perovskite deposition procedure is provided in the Supporting Information. (TMS)_2_S is a well‐established sulfur precursor for PbS nanocrystal synthesis, where sulfide is released by cleavage of the Si–S bond.^[^ ^26^ ^]^ Lead salts, such as Pb(PyA)_2_, then reacts with the in situ‐generated trimethylsilyl hydrosulfide (HSSiMe_3_) to give transient Pb(SH)_2_–solvent complexes, which condense during annealing to form the PbS layer. A slight color change from transparent to pale brown was observed for the entire film, suggesting the presence of the in situ reaction (Figure S2, Supporting Information).

X‐ray Photoelectron Spectroscopy (XPS) was first employed to characterize the surface composition. Distinct Pb^2+^ and S^2−^ peaks can be identified after the in situ reaction (Figure 1b,c), confirming the formation of PbS. Time‐of‐Flight Secondary Ion Mass Spectrometry (ToF‐SIMS) results further supported that the signals of lead and sulfide are predominantly concentrated at the surface (Figure S3, Supporting Information). The thicknesses of the films treated with the optimal concentration as mentioned above and 10‐fold higher concentrations were measured using a profilometer (Figure S4, Supporting Information). Since the thicknesses of the bare film and the film treated with the optimal concentrations were very close, the thickness of the PbS layer deposited using 10‐fold higher concentration (≈35 nm) is used to estimate the thickness of the optimal PbS layer to be ≈3 nm. The cross‐sectional Transmission Electron Microscopy (TEM) image of a PbS layer formed through the same recipe on a silicon substrate also revealed a thickness of ≈2 nm, confirming its ultra‐thin nature (Figure 1d). The Energy Dispersive X‐ray (EDX) mapping presented a clear layer of signal from PbS interposed by the fullerene and the silicon substrate (Figure 1e).

Additionally, quantitative analysis from EDX spectra reveals a trace amount of lead (≈0.7%) in the film with the PbS layer (Figure S5, Supporting Information), agreeing with the XPS and ToF‐SIMS results. Detailed lead content analysis is discussed in Note S1 (Supporting Information). Combining XPS, ToF‐SIMS, and EDX spectra, the lead content in the near‐surface region is estimated to be ≈1%, and only ≈ 0.1 wt% in the full film stack. Despite the low lead content in the thin film, we assessed risk relative to established environmental regulations. First, the Pb is present as PbS, whose aqueous solubility is extremely low (solubility product constant, K_sp_ ≈ 3 × 10^−28^). In a worst‐case scenario, assuming 1 cm^3^ of water dissolves per cm^2^ of perovskite film, the lead concentration would be <1 ppm, significantly below the US Environmental Protection Agency (EPA) limit of 5.0 mg/L. When the entire module mass (including glass and encapsulant) is considered, the lead content remains under the 0.1 wt% threshold specified by the EU Restriction of Hazardous Substances (RoHS) directive. This negligible lead content suggests that the environmental impact of the device is minimal.

### 2.2 Crystalline Structure Characterization

**Figure 2:**
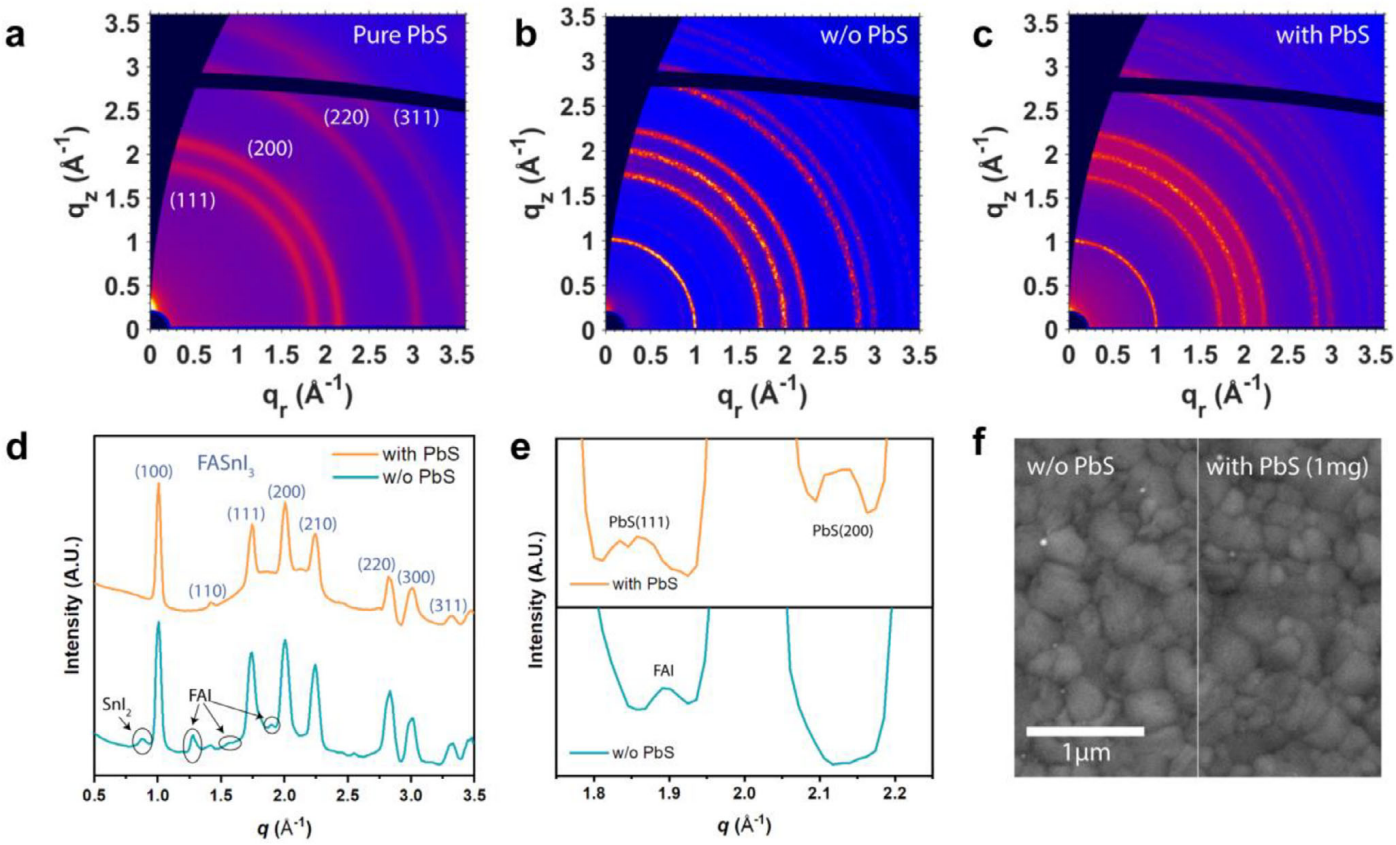
GIWAXS diffraction patterns by synchrotron microbeam of a) the pure PbS film on a silicon substrate, b) the film without the PbS layer, and c) the film with the PbS layer. d) Line‐cut profiles, integrating over the azimuthal angle, of the GIWAXS diffraction patterns (log‐scale of the intensity profile is used to enhance the contrast of the weak peaks). e) Enlarged line‐cut profiles of the GIWAXS diffraction patterns. f) SEM images of the perovskite film without and with the PbS layer.

After confirming the presence of lead and sulfur at the surface, we then employed Grazing‐Incidence Wide‐Angle X‐Ray Scattering (GIWAXS) to examine the crystal structure of the top layer. **Figure** **2**a presents the synchrotron‐based GIWAXS diffraction pattern of a pure PbS film, displaying diffraction rings at scattering vectors **q** of 1.86, 2.16, 3.03, and 3.56 Å^−1^, corresponding to the (111), (200), (220), and (311) planes of the cubic PbS phase with a lattice constant of *a* = 5.83 Å, consistent with previously reported values.^[^ ^27^ ^]^ Figure 2b,c presents the GIWAXS patterns of the FASnI_3_ perovskite films without and with the PbS layer, respectively, while the corresponding intensity profiles, integrated over the polar angle, are shown in Figure 2d. Characteristic diffraction peaks of the α‐phase FASnI_3_ perovskite are clearly observed in both patterns.

Furthermore, the perovskite film without the PbS layer exhibits weak additional diffraction peaks, attributable to secondary phases such as SnI_2_ and FAI. This suggests the onset of perovskite degradation in the unprotected film. The in‐house GIWAXS measurements also illustrated the emergence of the FAI peaks upon degradation (Figure S6, Supporting Information). In contrast, these secondary peaks are absent in the film with the PbS layer. Instead, the (111) and (200) diffraction peaks of PbS are clearly identifiable in Figure 2e, confirming the formation of an ultra‐thin layer of PbS on top of the perovskite film. In addition, the lattice constant of the PbS layer, when grown on the perovskite film, is calculated to be 5.88 Å, slightly larger than that of the pure PbS film (5.83 Å). This lattice expansion is likely due to the interaction with the underlying perovskite film, which possesses a larger lattice constant of ≈6.28 Å (≈6% larger). The nature of this interaction will be further explored in subsequent sections.

To further probe the crystallinity at different depths within the films, GIWAXS images were acquired at incident angles ranging from 0.05° to 0.50°. Notably, the perovskite film with the PbS layer consistently exhibits higher diffraction peak intensities across all incident angles except at 0.05° (Figure S7, Supporting Information), indicating enhanced crystallinity throughout the bulk of the film. The comparable intensity at 0.05° is likely due to X‐ray absorption by the PbS layer at grazing incidence, where the beam path within the PbS layer is maximized. Supporting this, increasing the PbS layer thickness leads to a further decrease in scattering intensity, as shown in Figure S8 (Supporting Information). Scanning Electron Microscopy (SEM) results further support these observations. The SEM images of the films with and without the PbS layer show comparable grain sizes (Figure 2f). Encouragingly, the treated film exhibits fewer pinholes, reflecting the protective role of the PbS layer against oxidation. When the concentration of the processing solution is increased (5 mg mL^−1^ of Pb(PyA)_2_ and 15 mg mL^−1^ of (TMS)_2_S), grain features disappear, demonstrating the formation of a thicker PbS layer (Figure S9, Supporting Information).

### 2.3 Electronic Properties

**Figure 3:**
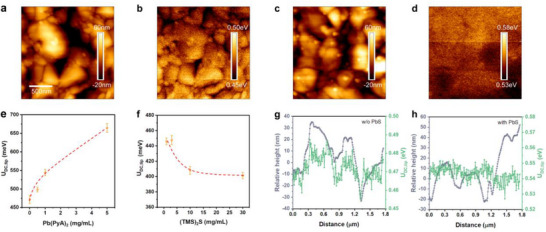
a) AFM and b) KPFM images of the film without the PbS layer. c) AFM and d) KPFM images of the film with the PbS layer. e) The impact of various concentrations of the lead salt, keeping (TMS)_2_S concentration at 3 mg mL^−1^, on *U*_DC,tip_. f) The impact of various concentrations of the sulfide source, keeping Pb(PyA)_2_ concentration at 0.5 mg mL^−1^, on *U*_DC,tip_. Line‐cut profiles of the diagonal of the tip‐sample potential, *U*_DC,tip_, of g) the film without the PbS layer, and h) the film with the PbS layer.

The impact of the PbS layer on the electronic properties of the perovskite films was investigated by Kelvin Probe Force Microscopy (KPFM). In KPFM, the tip‐sample potential (*U*_DC,tip_) is related to the contact potential difference (*U*_CPD_) as *U*_CPD_ = −*U*_DC,tip_, where a higher *U*_DC,tip_ value corresponds to a lower work function of the sample. The Atomic Force Microscopy (AFM) images of the film without and with the PbS layer are shown in **Figure** **3**a,c. The corresponding average *U*_DC,tip_ values obtained from Figure 3b,d for the film without and with the PbS layer are 471.7 and 544.6 meV, respectively. The higher *U*_DC,tip_ for the film with the PbS layer corresponds to a lower work function, indicating a more n‐type surface. This conclusion is further substantiated by Ultraviolet Photoelectron Spectroscopy (UPS) data (Figure S10, Supporting Information), which reveals an upward shift of the perovskite Fermi level toward the conduction band minimum (CBM) by ≈0.07 eV with the PbS capping layer.

The formation mechanism of the n‐type PbS layer was explored by modulating the relative concentrations of Pb(PyA)_2_ and (TMS)_2_S. Literature reports indicate that a slight lead excess in PbS leads to n‐type behavior, whereas a slight sulfur excess results in p‐type behavior.^[^ ^28^, ^29^ ^]^ As the concentration of Pb(PyA)_2_ was incrementally increased from 0 to 5 mg mL^−1^, the measured *U*_DC,tip_ exhibited a progressive rise from 471.7 to 664.5 meV, signifying a transition toward a more n‐type surface (Figure 3e). And vice versa, increasing the sulfur abundance by increasing the concentration of (TMS)_2_S from 0 to 30 mg mL^−1^ caused *U*_DC,tip_ to decrease from 445.9 to 401.2 meV, manifesting a more p‐type surface (Figure 3f). The energy diagram, derived from the UPS spectra, is shown in Figure S10 (Supporting Information). A more n‐type PbS layer facilitates efficient charge extraction, contributing to the observed improved *V*_OC_ as detailed in the following section.

Furthermore, while the Atomic Force Microscopy (AFM) images of both films show similar topography (Figure 3a,c), the KPFM images reveal notable differences in the homogeneity of the surface potential (Figure 3b,d). For the film without the PbS layer, a clear correlation between the *U*_DC,tip_ and relative height is observed from the line‐cut profile along the diagonal of the KPFM image (Figure 3g), suggesting a higher work function at grain boundaries compared to grain interiors. The observed p‐doped grain boundary signifies stronger oxidation than grain interiors, consistent with previous computational predictions.^[^ ^30^ ^]^ Such potential variations can promote p‐type recombination and charge accumulation,^[^ ^31^ ^]^ which are detrimental to *V*_OC_, as discussed in the later section. In contrast, the film with the PbS layer exhibits much less potential variation in the line‐cut profile (Figure 3h). To further investigate the relationship between surface roughness and surface potential, the correlation coefficients between the height measured by AFM and the potential measured by KPFM are calculated. The film with the PbS layer shows a markedly lower correlation coefficient (0.165) than that of the film without the PbS layer (0.758) (Figure S11, Supporting Information), further corroborating the enhanced homogeneity of the surface potential in the presence of the PbS layer.

### 2.4 Stability Improvement with the PbS Capping Layer

**Figure 4:**
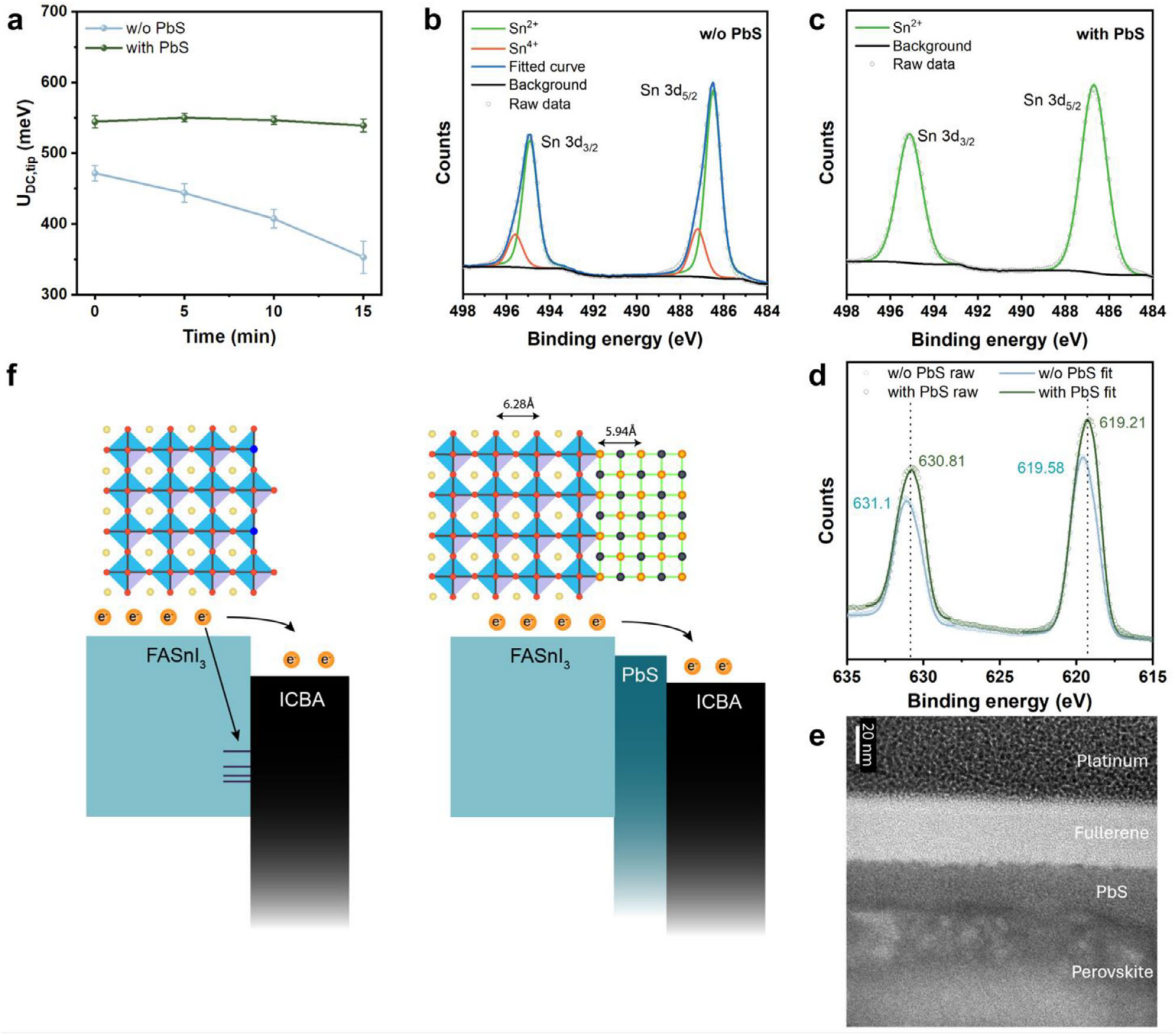
a) Time evolution of the statistical distributions of *U*_DC,tip_ of KPFM images of the film with and without the PbS layer over an area of 2 × 2 µm^2^. Sn 3d XPS spectra of b) the film without and c) the film with the PbS layer. d) XPS spectra of the I 3d peaks of both films. e) Cross‐sectional TEM image of the perovskite/PbS/ICBA interface. f) Schematics of the proposed mechanism of the in situ grew PbS layer.

To further investigate the air stability of the perovskite films, we monitored the temporal evolution of KPFM images for the films stored in ambient air (Figure S12, Supporting Information). The film without the PbS layer exhibited a clear decrease in *U*_DC,tip_ from 471.7 to 352.9 meV within 15 min, attributed to the rapid oxidation of tin(II) (**Figure** **4**a; Figure S13a, Supporting Information). The full‐width‐at‐half‐maximum (FWHM) of the work function distribution also increased from 25.7 to 54.1 meV, indicating a broader distribution and more inhomogeneous work function profile. This inhomogeneity arises from the uneven oxidation rates across the grain boundaries, as discussed earlier. In stark contrast, the film with the PbS layer demonstrated remarkable stability, with *U*_DC,tip_ changing only slightly from 544.6 to 539.1 meV over the same period (Figure 4a; Figure S13b, Supporting Information). The FWHM also remained nearly constant, increasing marginally from 20.2 to 21.7 meV. These KPFM results highlight the protective role of the PbS layer, which effectively mitigates oxidation and maintains a stable and uniform surface potential.

The passivation ability of the PbS layer was further studied through detailed analysis of specific XPS spectrum regions. Tin‐based perovskites are known to be susceptible to oxidation, leading to a significant population of tin(II) vacancies.^[^ ^30^ ^]^ By analyzing the Sn 3d core‐level spectra (Figure 4b,c), the ratio of Sn^4+^:Sn^2+^ ions was estimated to be 20% for the perovskite film without the PbS layer, while the Sn^2+^ content is negligible in the film with PbS protection. We then monitored oxidation under ambient air for 5, 20, and 60 min (Figure S14, Supporting Information). In the uncapped film, the Sn^4+^ fraction rose to 43%, 60%, and finally 91%, indicating rapid oxidation. By contrast, the PbS‑protected film retained a negligible Sn^4+^ level throughout the 60 min exposure, demonstrating that the ultrathin PbS layer effectively suppresses surface oxidation. This reduction in Sn^4+^ content demonstrates the protective role of the PbS layer in mitigating oxidation. The enhanced stability is attributed to the inherent chemical inertness of PbS in ambient environments.^[^ ^32^ ^]^ Given the similar electronegativity of lead and tin, the shifts in the XPS peaks of iodide can be attributed to changes in coordination number. The I 3d_3/2_ and I 3d_5/2_ peaks shifted from 631.1 to 630.8 eV and from 619.6 to 619.2 eV, respectively, indicating a reduction in the oxidation state of the iodide atoms (Figure 4d). This interaction between the lead atoms in the PbS layer and the underlying tin‐based perovskite explains the observed shifts in the XPS peaks of iodide.

To visualize the morphology and interfacial quality of the PbS layer between perovskite and ICBA, cross‐sectional Transmission Electron Microscopy (TEM) was performed, with a platinum protective layer deposited on top (Figure 4e). To compensate for focused ion‐beam (FIB) damage during TEM sample preparation, we deposited a PbS layer at ten times the standard concentration. The TEM image reveals that the PbS layer forms a compact and uniform interfacial layer between the perovskite and ICBA. The PbS layer exhibits excellent adhesion to both the perovskite and ICBA layers, with no visible voids or discontinuities. The uniformity and compactness of the PbS layer highlight its structural integrity as an interfacial layer. The functions of the PbS layer are summarized in Figure 4f. By carefully tuning the ratio between Pb(PyA)_2_ and (TMS)_2_S, we engineered the PbS layer to function as an n‐type material, creating an effective charge extraction channel. Furthermore, the air‐stable PbS layer serves as a robust protective barrier against oxidation and moisture, enhancing the overall stability of the perovskite film in ambient conditions.

### 2.5 Device Characteristics and Device Stability

**Figure 5:**
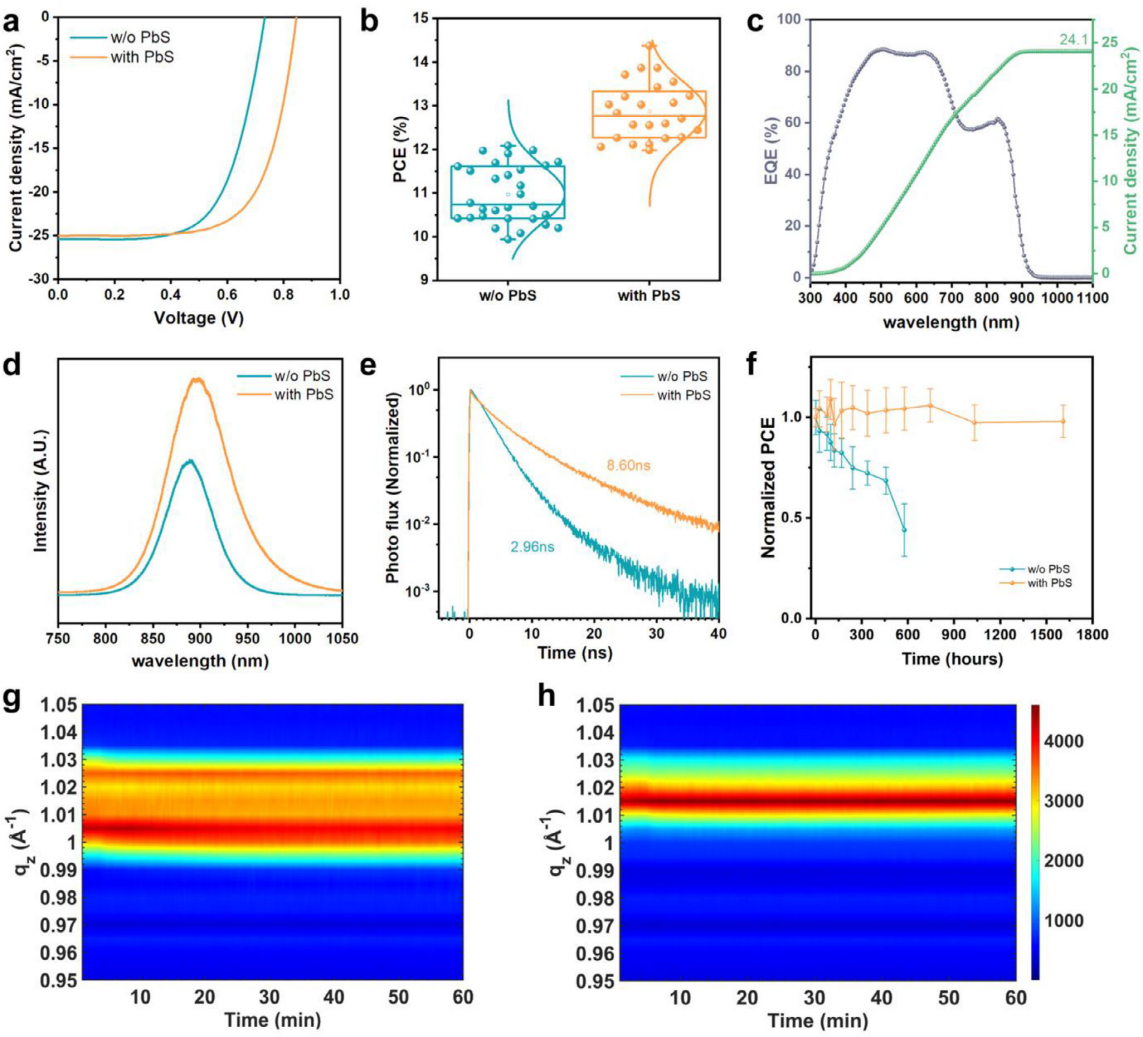
a) *J–V* curves and b) PCE comparison of the solar cell devices fabricated with and without the PbS layer under AM1.5G solar 100 mW cm^−2^. c) EQE spectrum of the solar cell devices fabricated with the PbS layer. d) Steady‐state PL and e) TRPL measurements of the perovskite films with and without the PbS layer. f) Long‐term stability test of the encapsulated solar cell devices fabricated with and without the PbS layer stored in a N_2_ atmosphere. Evolution of the line‐cuts of the GIWAXS intensity profiles along the out‐of‐plane direction of the perovskite films g) without and (hi) with the PbS layer.

To evaluate the impact of the PbS layer on device performance and stability, we fabricated solar cell devices with the structure ITO/PEDOT:PSS/Perovskite/PbS/ICBA/Ag. Compared to the control devices, those incorporating the PbS layer demonstrated significant improvements in *V*_OC_ and PCE (**Figure** **5**a,b; Figure S15, Supporting Information). The champion device with the PbS layer achieved a *V*_OC_ of 0.845 V, representing a 0.118 V enhancement over the control cell, and the PCE increased from 12.0% to 14.4%. The significant *V*_OC_ improvement originates from the suppression of p‐type recombination centers and charge accumulation at the grain boundaries by the PbS layer. The external quantum efficiency (EQE) spectra revealed an integrated *Jsc* of 24.1 mA cm^−^^2^ (Figure 5c), closely matching the *Jsc* of 25.0 mA cm^−2^ obtained from current density‐voltage (*J–V*) characterization. Notably, devices coated solely with Pb(PyA)_2_ or (TMS)_2_S did not exhibit comparable improvements in *V*_OC_, underscoring the critical role of the PbS capping layer in enhancing device performance (Figure S17, Supporting Information).

Additionally, steady‐state photoluminescence (PL) and time‐resolved photoluminescence (TRPL) spectra were collected to analyze the recombination dynamics of the devices. The steady‐state PL spectra showed a stronger emission peak at ≈880 nm for the device with the PbS layer, suggesting significant suppression of non‐radiative recombination (Figure 5d). A red‐shifted shoulder extending beyond 920 nm is also observed for the film with PbS layer. This feature is attributed to shallow tail states generated by the slight lattice mismatch and interfacial strain between FASnI_3_ and PbS. An analogous red shift of the PL peak has been observed along with lattice strain.^[^ ^33^ ^]^ Despite the appearance of shallow traps, deep traps are sufficiently passivated, yielding a stronger PL signal. Consistent with these findings, the carrier lifetime extracted from the TRPL spectra increased from 2.96 ns for the control cell to 8.60 ns for the cell with the PbS layer. (Figure 5e).

The storage stability of 18 solar cell devices was tracked under a nitrogen‐filled glovebox to assess the protective role of the PbS layer (Figure 5f). Control devices exhibited a rapid decline in performance, retaining half of their original PCE within 600 h. In contrast, devices with the PbS layer maintained nearly their initial PCE after 1600 h, demonstrating exceptional stability. These findings align with the stable contact potential observed in the KPFM measurements, underscoring the protective efficacy of the PbS layer. (Figure 4a).

To further investigate operational stability under realistic conditions, we conducted *operando* GIWAXS measurements under constant AM1.5G illumination and a constant voltage near the maximum power point (0.6 V) in ambient air (Figure S18, Supporting Information). The *operando* measurements simulated real‐world operating conditions and captured potential structural changes. For the device without the PbS layer, the (100) peak intensity initially increased slightly within the first 20 min, likely due to light‐induced lattice expansion that relaxed local strain and improved lattice order.^[^ ^34^ ^]^ However, the peak intensity subsequently decreased over the next 40 min, resulting in a net reduction of 7.4% after 60 min of operation (Figure 5g). This weakening of lattice order is attributed to perovskite degradation under the combined stress of voltage and illumination. Notably, the initial increase in peak intensity, which was more pronounced in in situ GIWAXS measurements under vacuum (Figure S19, Supporting Information), suggesting that photo‐induced lattice strengthening occurs concurrently with degradation. Consequently, the actual degradation rate for the control device in ambient conditions must be underestimated in *operando* studies. In contrast, the (100) peak intensity of the device with the PbS layer remained relatively stable, even showing a 3.6% increase after the same operation period (Figure 5h). This reflects the robust structural stability of the perovskite film under operational stresses when protected by the PbS layer. The PbS layer effectively shields the perovskite from oxidation and moisture, preserving the integrity of the solar cell devices. In summary, the PbS layer not only enhances the optoelectronic properties of perovskite solar cells by reducing trap density and suppressing non‐radiative recombination but also provides exceptional protection against degradation under both storage and operational conditions.

## 3 Conclusion

In conclusion, this study demonstrates the successful fabrication of stable and efficient tin‐based perovskite solar cells by incorporating an in situ‐grown ultra‐thin PbS layer using Pb(PyA)_2_ and (TMS)_2_S. The inorganic PbS capping layer serves as a robust protective barrier, effectively shielding the perovskite from oxidation and moisture, thereby preserving the structural and optoelectronic integrity of the tin‐based perovskite. Beyond its protective role, the PbS layer can be precisely engineered into a suitable n‐type semiconductor material by tuning the ratio between Pb(PyA)_2_ and (TMS)_2_S, which significantly enhances charge extraction from the perovskite to the ICBA electron transport layer (ETL). Furthermore, the interaction between PbS and FASnI_3_ enables the PbS layer to grow on the perovskite surface, reconstructing the interface and reducing interfacial defects. This results in a substantial suppression of non‐radiative recombination, further improving device performance. As a result of these advancements, the PCE of the champion solar cell increased to 14.4%, accompanied by a remarkable enhancement in open‐circuit voltage. Moreover, the devices exhibited excellent stability, with negligible performance decay after 1600 h of storage in a N_2_ atmosphere. This work establishes the PbS layer as a highly promising strategy for overcoming the intrinsic instability challenges of tin‐based perovskite solar cells without compromising efficiency, a common trade‐off in other stabilization methods. By effectively addressing both stability and charge transport limitations through this dual‐functional approach, this research opens a promising and scalable avenue for realizing the full potential of tin‐based perovskite photovoltaics and unlocking their immense potential for environmentally friendly and commercially viable solar energy.

## Conflict of Interest

The authors declare no conflict of interest.

## Author Contributions

P.F.C., H.Z., and X.L. conceived the study, designed the experiments, and conducted the primary characterizations. Y.L. and J.W. performed the ToF‐SIMS measurements. H.C. assisted with material synthesis. X.W. and N.Z. performed the TRPL measurements. C.J.S. assisted with the operando GIWAXS measurements, and J.M.L. assisted with the synchrotron microbeam GIWAXS measurements. All authors contributed to the manuscript's review and editing.

## Supporting information



Supporting Information

## Data Availability

The data that support the findings of this study are available in the supplementary material of this article.
