# Supplementary material for: Ultra‐Thin Lead Sulfide Capping for Stabilizing Tin Halide Perovskite Solar Cells
Source: Small. 2025 Aug 23;21(40):e06340. doi: 10.1002/smll.202506340 (PMC12508707; doi:10.1002/smll.202506340)
Supplement: Supplementary file 1 — Supporting Information [file SMLL-21-e06340-s001.docx]

Supporting Information

Lead Sulfide Capping for Enhanced Charge Extraction in Tin Halide Perovskite Solar Cells

*Pok Fung Chan^1^, Yongmin Luo^2^, Haiyang Cheng^1^, Xuezhou Wang^3^, Ni Zhao^3^, Jiaying Wu^2^, Chun-Jen Su^4^, Jhih-Min Lin^4^, Hengkai Zhang^1^ and Xinhui Lu^1^**^,^**

^1^Department of Physics, The Chinese University of Hong Kong, New Territories 999077, Hong Kong SAR, China

^2^Advanced Materials Thrust, Function Hub, Hong Kong University of Science and Technology (Guangzhou), Guangzhou, China

^3^Department of Electronic Engineering, The Chinese University of Hong Kong, New Territories, Hong Kong SAR, China

^4^National Synchrotron Radiation Research Center, Hsinchu Science Park, Hsinchu 30076, Taiwan

**Materials**

Formamidinium iodide (FAI), methylammonium iodide (MAI), and iso-Butylammonium iodide were purchased from Greatcell Solar; ITO glass was purchased from Advanced Election Technology Co. Ltd; Poly(3,4-ethylenedioxythiophene) polystyrene sulfonate (PEDOT:PSS, Al 4083) was purchased from Ossila Ltd;. Indene-C60 Bisadduct (ICBA) was purchased from 1-Material Inc. (6,6)-Phenyl-C61-butyric acid methyl ester (PCBM) was purchased from Xi'an Polymer Light Technology Corp. All other chemical materials were purchased from Sigma-Aldrich and used as received unless stated otherwise.

**Synthesis of Pb(PyA)_2_ powders**

Pb(PyA)_2_ powders were synthesized following a previously reported procedure[22] Briefly, an excess of picolinic acid was dissolved in pure ethanol to achieve a concentration of approximately 0.1 M. Lead(II) oxide (PbO), with a concentration less than half that of picolinic acid (i.e., <0.05 M), was subsequently added to the solution. Due to the insolubility of PbO in ethanol and the solubility of picolinic acid, excess picolinic acid could be effectively removed by ethanol washing after the reaction. The mixture of picolinic acid and PbO was heated at 85 °C for over 72 hours, during which the yellow PbO was completely converted into white Pb(PyA)_2_ powder. The resulting powder was then recrystallized using a mixed solvent system of ethanol and dimethylformamide (DMF). Finally, the purified Pb(PyA)_2_ powder was dried by heating at 60 °C under a nitrogen atmosphere.

**Device fabrication**

ITO substrates were sequentially rinsed by sonication in detergent, deionized (DI) water, acetone, and isopropanol for 30 min, respectively, and then dried under nitrogen gas before use. Cleaned ITO substrates were treated with ultraviolet-ozone for 15 min, followed by the deposition of a hole transporting layer of PEDOT:PSS by spin-coating the PEDOT:PSS solution at 4500 rpm for 30 s, and then annealed at 130 ºC for 30 min. The SnI_2_ precursor was prepared by dissolving I_2_ (1.0 M) in a mixture of DMF/DMSO with the volume ratio of 4:1, and then excess Sn powder was added, followed by a 30 min of vigorous shaking. The FASnI3 perovskite precursor was prepared by dissolving FAI (0.95 M), MAI (0.05 M), and SnF_2_ (0.1 M) into the SnI_2_ precursor, and then excess 6% 1.5 M PEAI was added into the precursor. The perovskite precursor was filtered to isolate the tin powder before use. The Pb(PyA)_2_ solution was prepared by dissolving 1 mg/mL Pb(PyA)_2_ in hexafluoro-2-propanol (HFP). The (TMS)_2_S solution was prepared by dissolving 3 mg/mL (TMS)_2_S in chlorobenzene (CB). The ICBA solution was prepared by dissolving 18 mg/mL ICBA in CB. The BCP solution was prepared by dissolving 0.5 mg/mL BCP in IPA. The perovskite film was deposited by spin-coating the perovskite precursor on the ITO/PEDOT:PSS substrate at 4500 rpm for 50 s, and 100 µL chlorobenzene containing 0.5 mg/ml PCBM was dripped onto the substrate at the 9th second from the start of spin-coating. The as-cast film was then annealed at 80 ºC for 30 min. For the PbS treatment, 30 uL of Pb(PyA)_2_ solution was first dripped onto the annealed perovskite film at 4000 rpm for 20 s, followed by the drip of (TMS)_2_S solution with the same spinning setting. The PbS coated film was then annealed at 80 ºC for 10 min. ICBA and BCP solutions were subsequently spin-coated at 2500 rpm and 4000 rpm for 20 s, respectively. Finally, a 100 nm Ag electrode was deposited by thermal evaporation.

**Characterizations**

GIWAXS measurements were carried out using a Xeuss 2.0 SAXS/WAXS laboratory beamline with a Cu X-ray source (8.05 keV, 1.54 Å) and a Pilatus 3R 300K detector. Operando GIWAXS experiments for the light stability tests of the perovskite devices were conducted at TLS 23A small- and wide-angle X-ray scattering (SWAXS) beamline and microbeam GIWAXS experiments were performed at TPS25A at the National Synchrotron Radiation Research Center (NSRRC), Hsinchu, Taiwan. The AM1.5G illumination is simulated with the Asahi Spectra, HAL-320. The J-V curves were measured by a Keysight B2901A source meter unit under an AM 1.5G solar simulator (SS-F5; Enli Technology, Taiwan), and the light intensity was calibrated using a standard silicon reference cell. X-ray photoelectron spectroscopy (XPS) and ultraviolet photoelectron spectroscopy (UPS) characterizations were performed at Shimadzu China Co. LTD, Guangzhou, 510656, China, using an AXIS Supra+, Kratos Analytical Inc. UV-Vis absorption spectra were taken on a Hitachi U-3501 ultraviolet/visible/near-infrared spectrophotometer. The perovskite surface morphology was characterized by a high-resolution field emission scanning electron microscopy (HR-FESEM) (FEI, Quanta 400). The cross-sectional TEM image was taken by the FEI Tecnai TF20 200kV FEG high resolution transmission electron microscrope. Photoluminescence measurements of perovskite ﬁlms on glass were conducted by using a Renishaw micro raman spectrometer, QONTOR, with an excitation laser of 633 nm. AFM and KPFM measurements were conducted with the Nikon Ti Inverted microscope bruker and the JPK atom force microscope with a single photon spectrometer. The probing tip was the ElectriMulti75-G probe from Budget Sensors. TRPL measurements were conducted using a photoluminescence spectrometer (iHR320, HORIBA). A 463 nm laser diode (DeltaDiode-470L, Horiba) as a pump source was used for photoexcitation with a frequency of 100 MHz. The TRPL spectrum was obtained by a high-sensitivity photon counting detector (TRPL-PPD900-MICOS, horiba). External quantum efficiency (EQE) of the solar cells was measured by a QE-R 3011 system (Enli Technology, Taiwan).


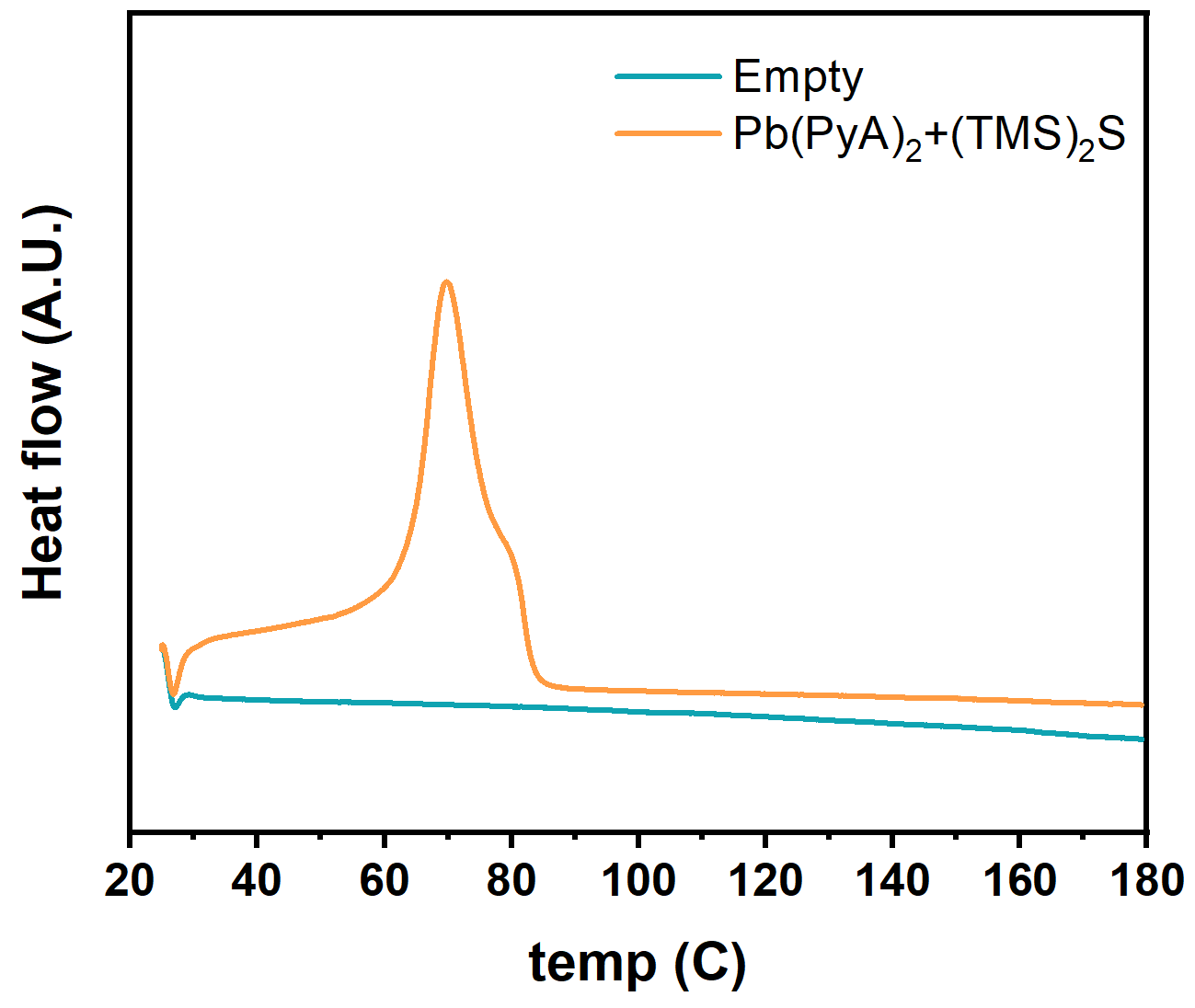


**Figure S1**. Differential Scanning Calorimetry (DSC) measurement results of the reaction between Pb(PyA)_2_ and (TMS)_2_S.


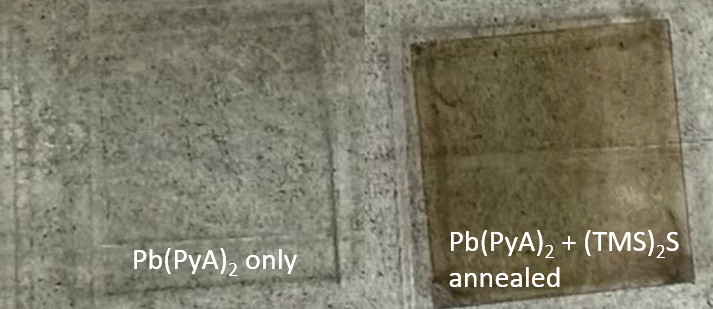


**Figure S2**. Photos of color change of the formation of PbS from the reaction between Pb(PyA)_2_ and (TMS)_2_S.


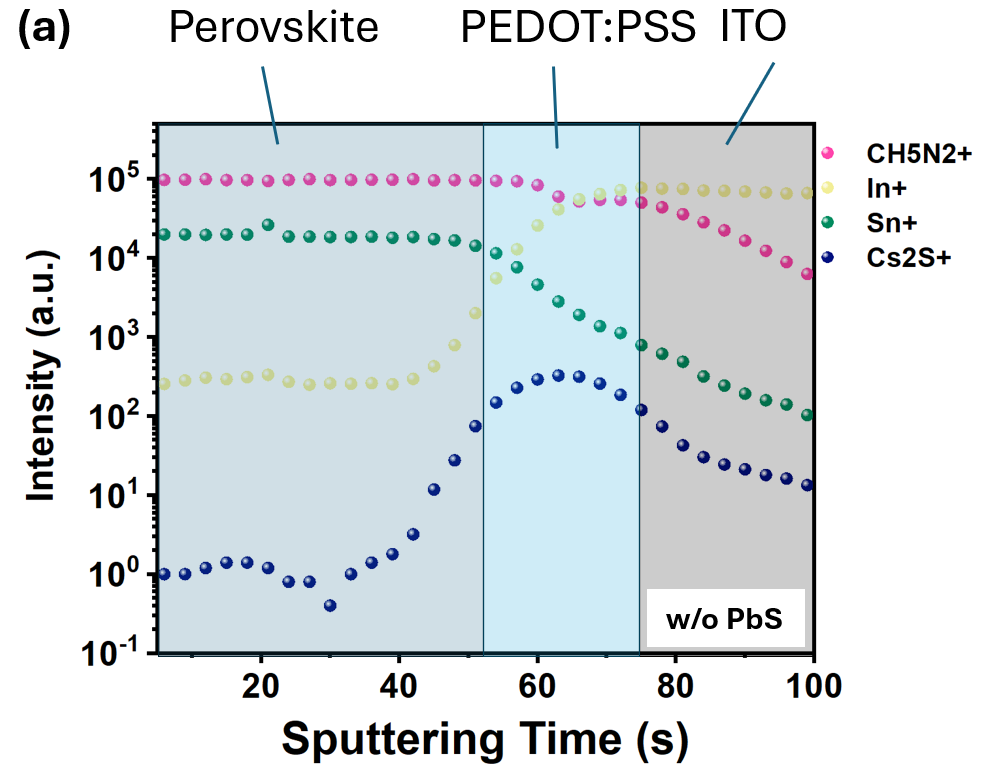


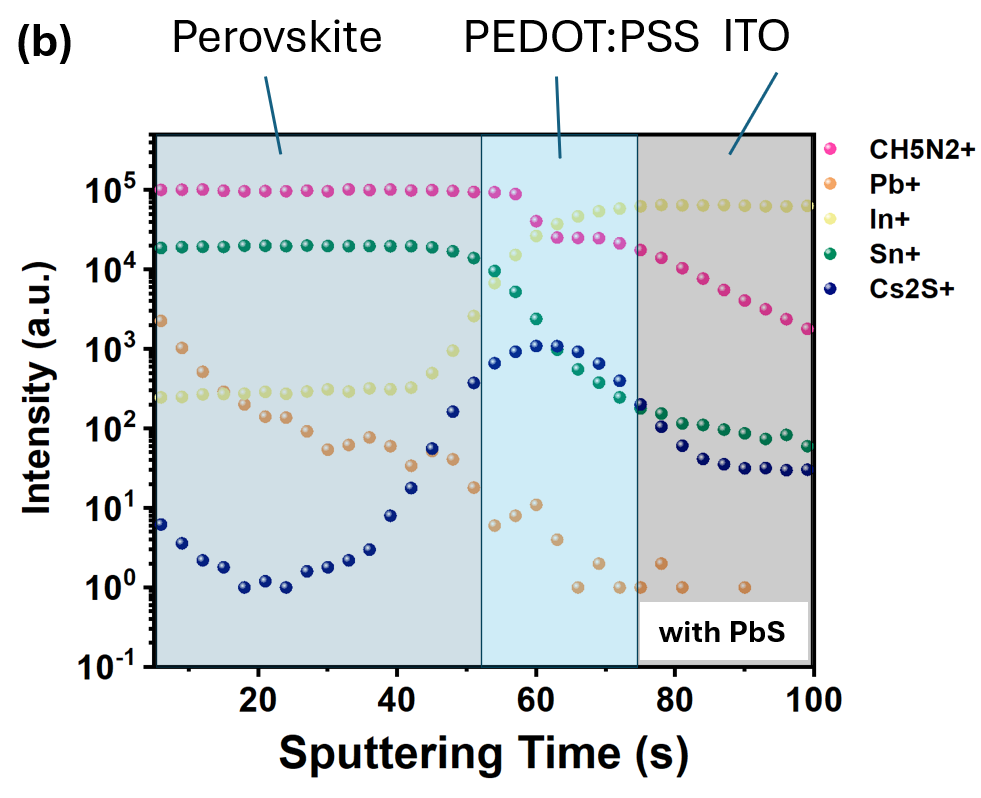


**Figure S3**. Time-of-flight Secondary Ion Mass (ToF-SIMS) spectra of the perovskite films (a) **without**, and (b) **with** the PbS layer.


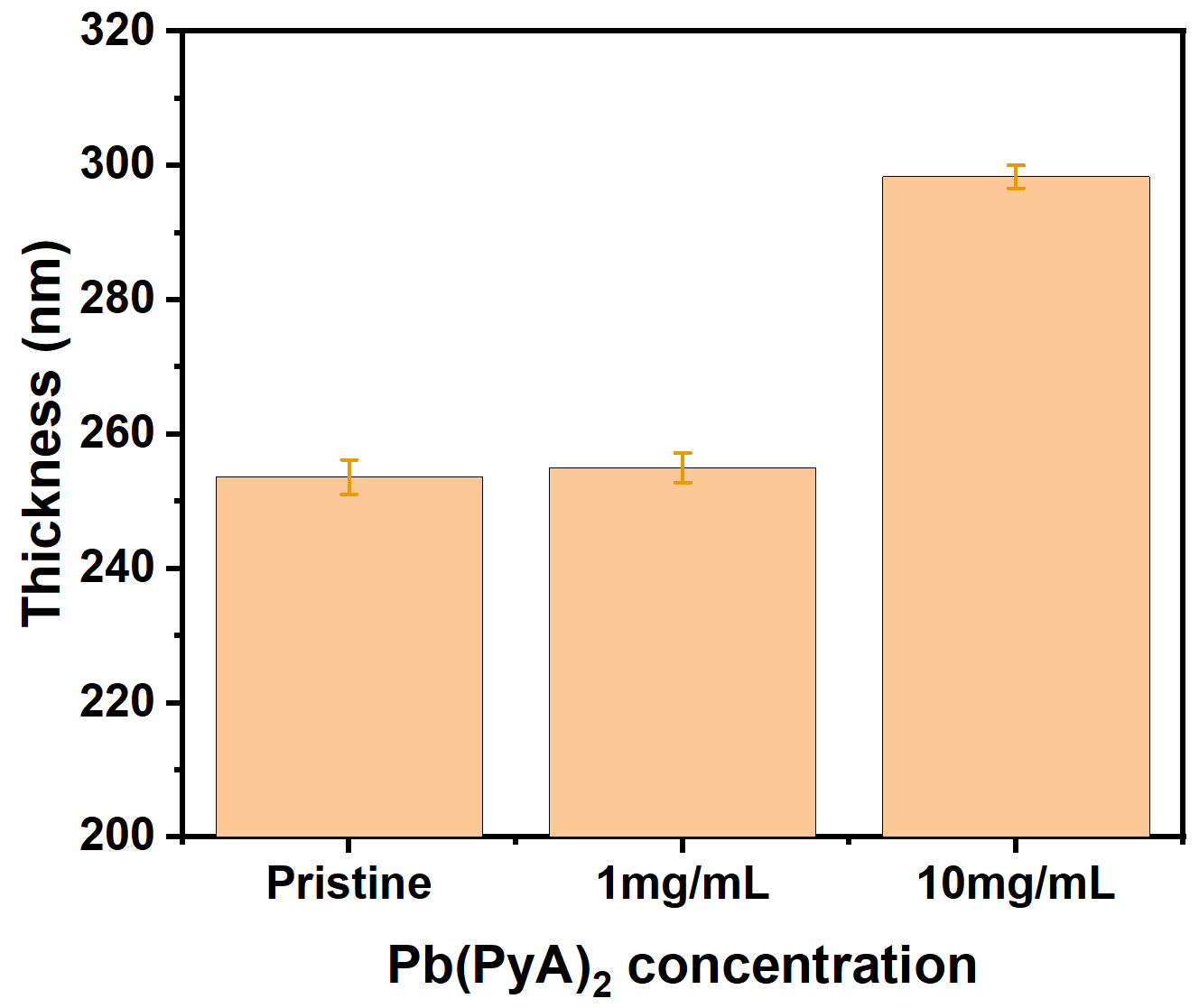


**Figure S4**. Thickness of the films coated with different Pb(PyA)_2_ concentrations (the concentration of (TMS)_2_S changes proportionately).


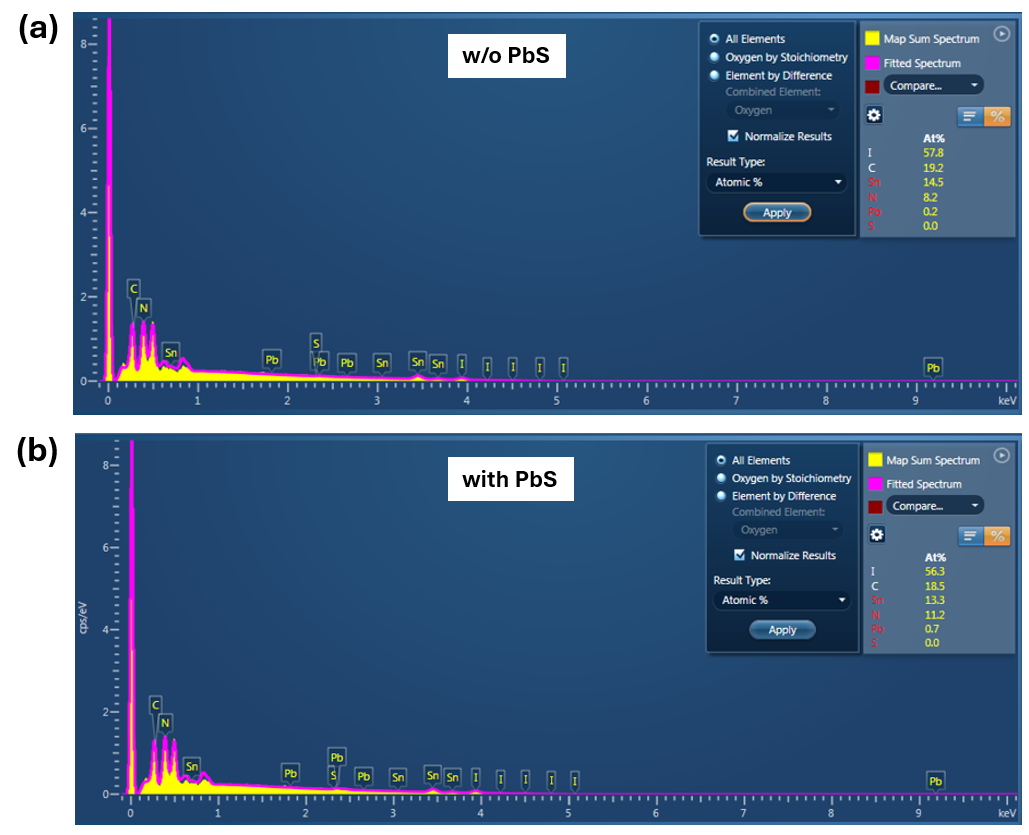


**Figure S5**. Energy Dispersive X-ray (EDX) spectra of the perovskite films (a) **without**, and (b) **with** the PbS layer.


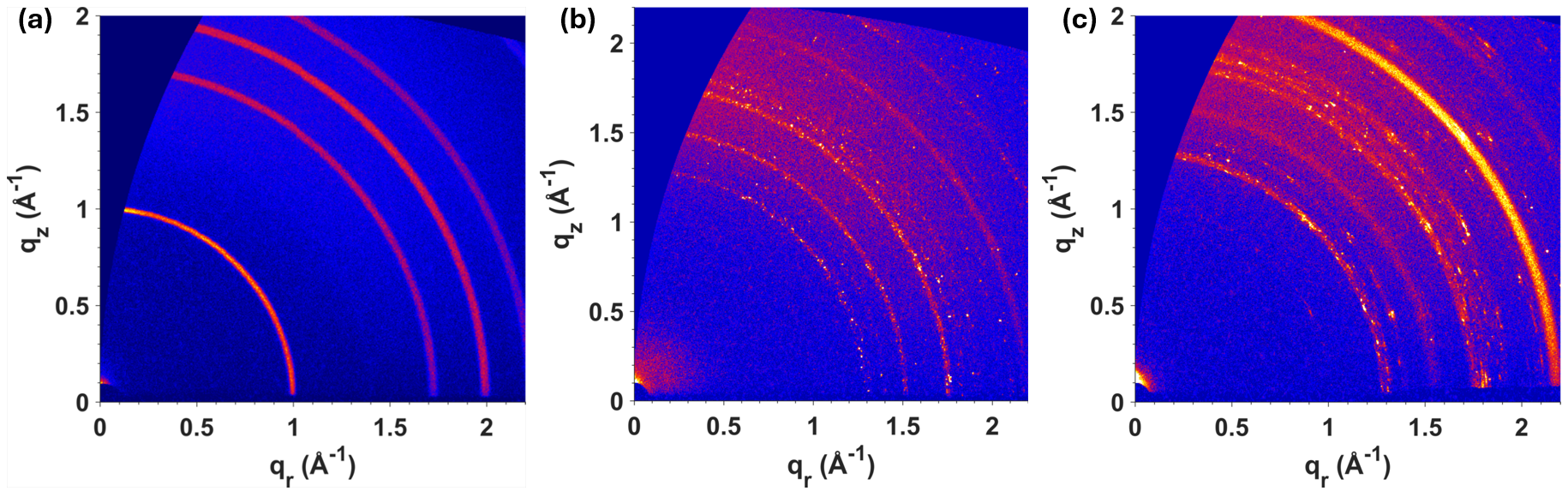


**Figure S6**. GIWAXS patterns of (a) the control perovskite film, (b) FAI spin-coated on a glass substrate, and (c) the perovskite film completely degraded after 1 week of storage in the ambient atmosphere.


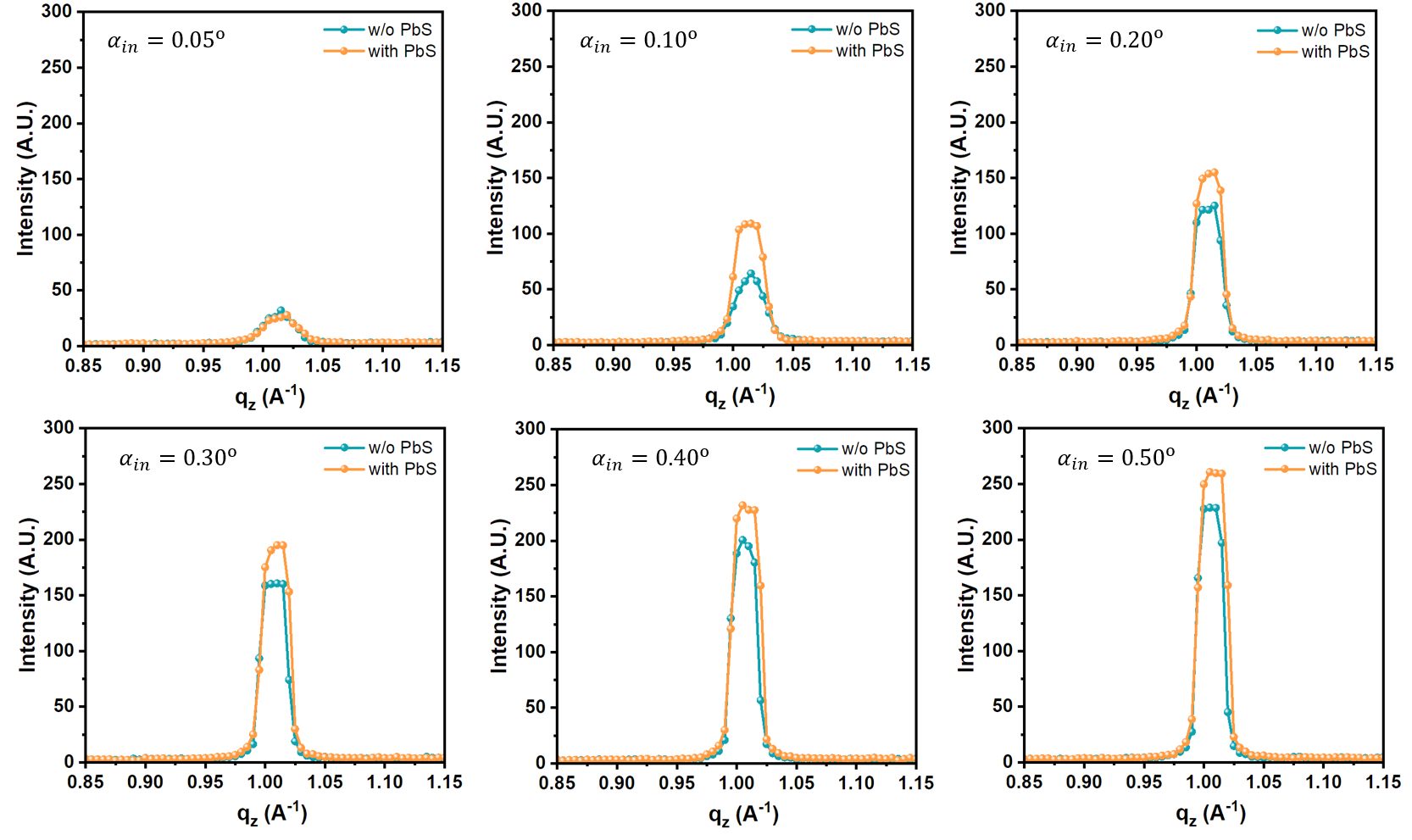


**Figure S7**. Line-cuts extracted from the GIWAXS patterns along the out-of-plane direction and the corresponding (100) peak intensity profiles comparison between the perovskite films with and without the PbS layer.


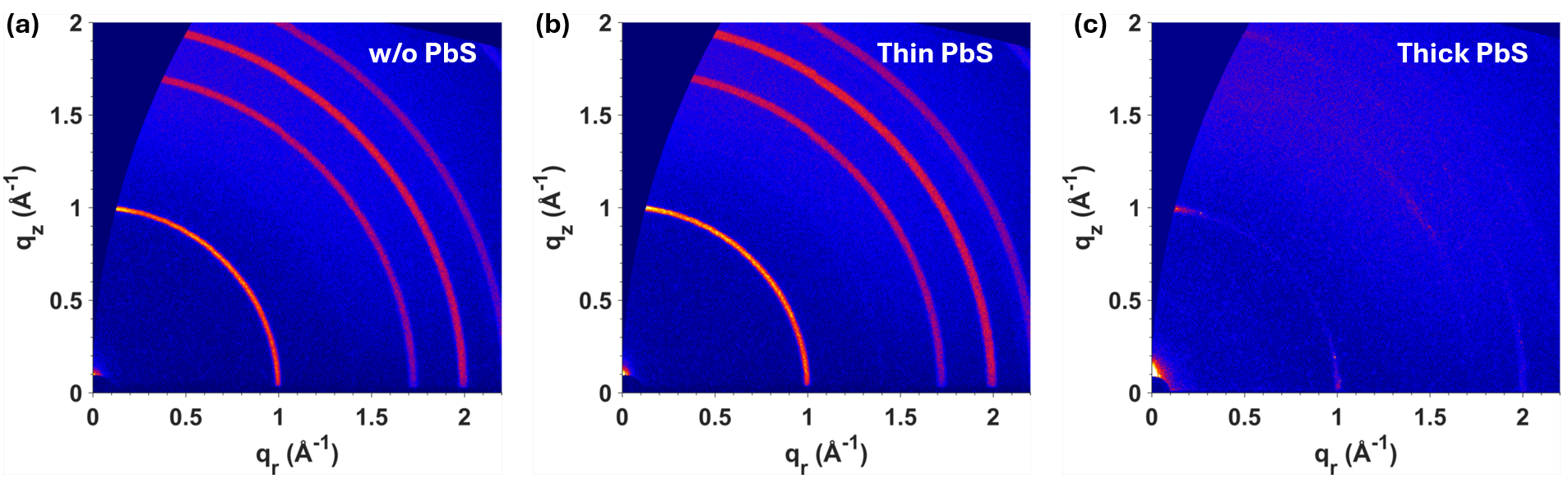


**Figure S8**. GIWAXS patterns of (a) the control perovskite film, (b) the film covered by a thin PbS layer (treated by 1mg/mL Pb(PyA)_2_ and 3mg/mL (TMS)_2_S), and (c) the film covered by a thick PbS layer (treated by 10mg/mL Pb(PyA)_2_ and 30mg/mL (TMS)_2_S)


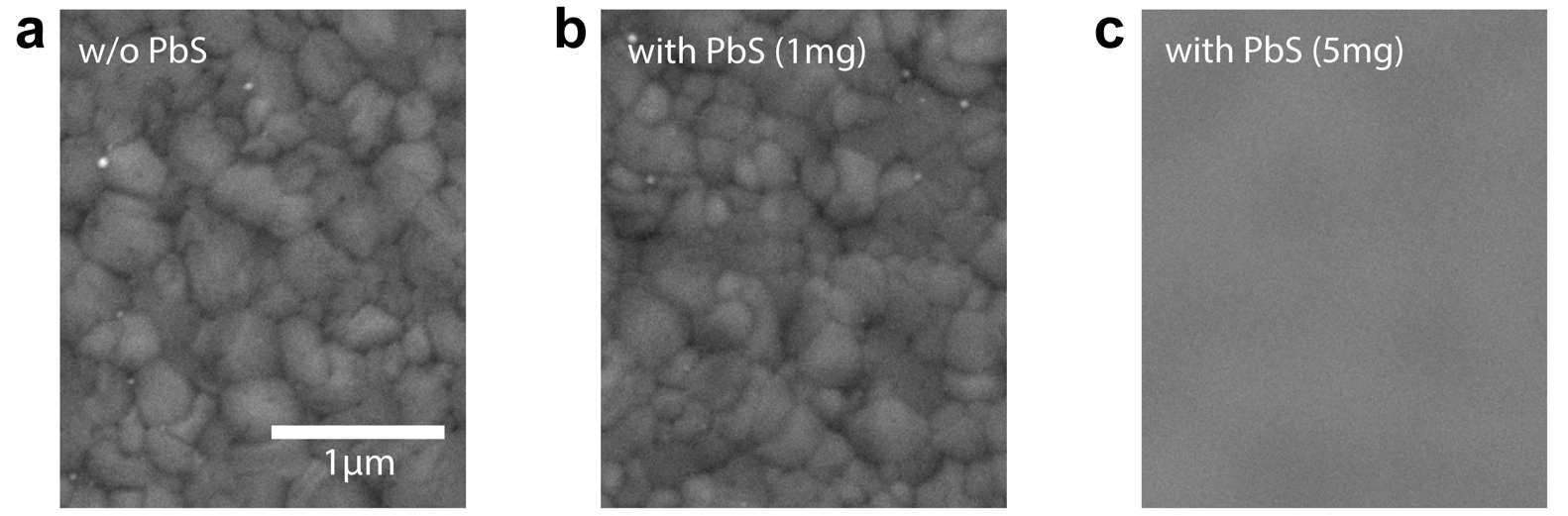


**Figure S9**. SEM images of the perovskite film with different thicknesses of the PbS layer deposited with various concentrations of Pb(PyA)2.


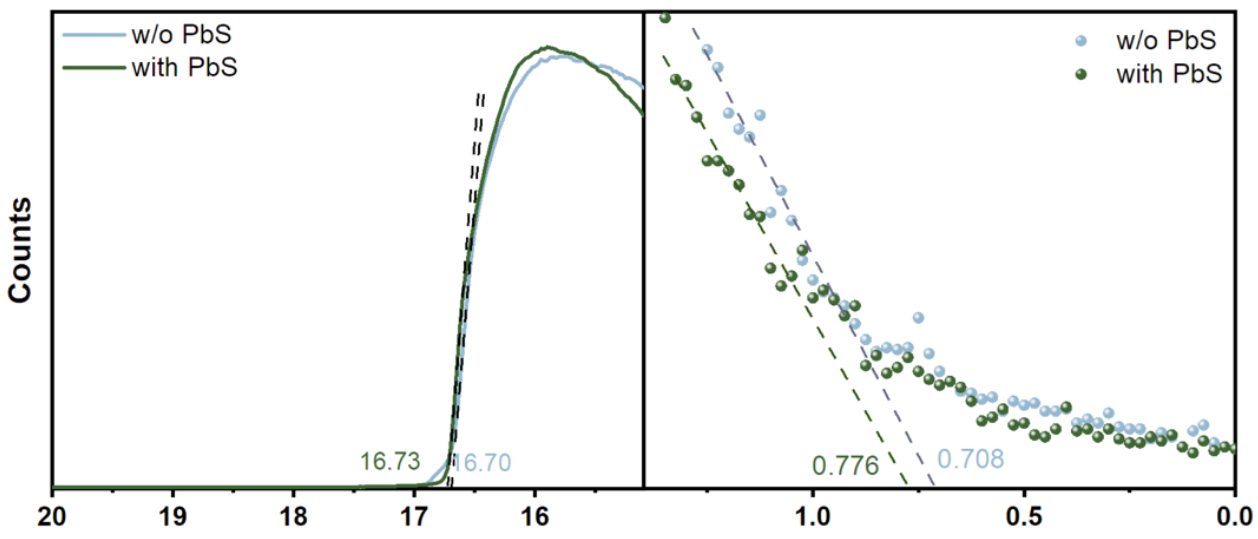


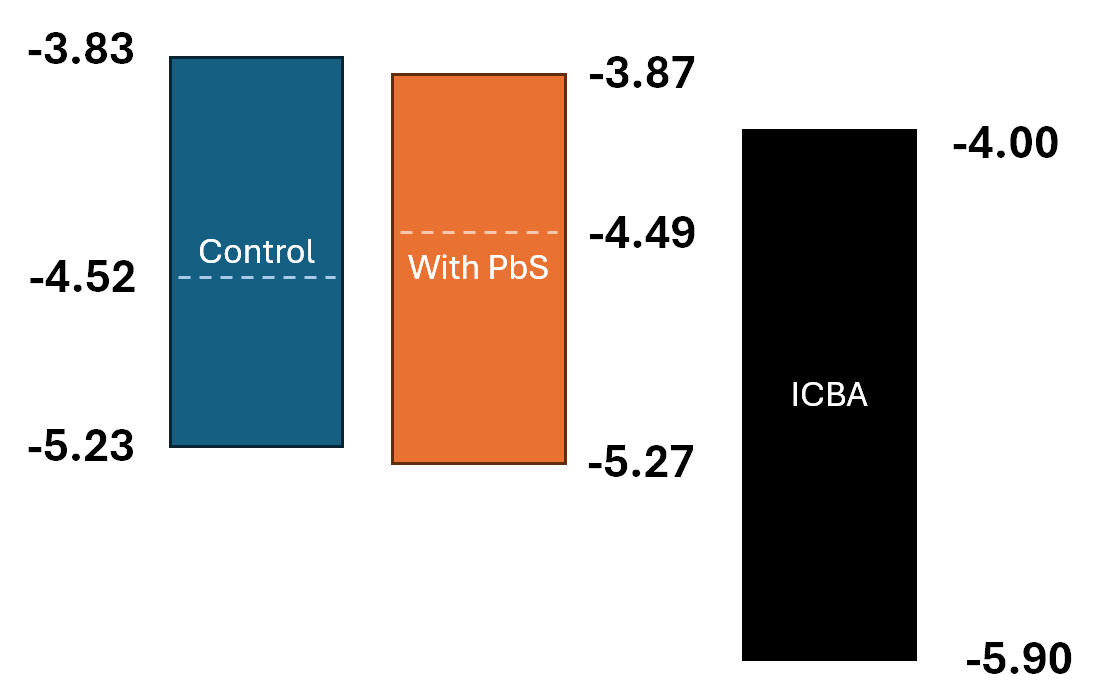


**Figure S10**. UPS spectra and the energy levels of the perovskite films **without** and **with** the PbS layer deduced from the UPS spectra.


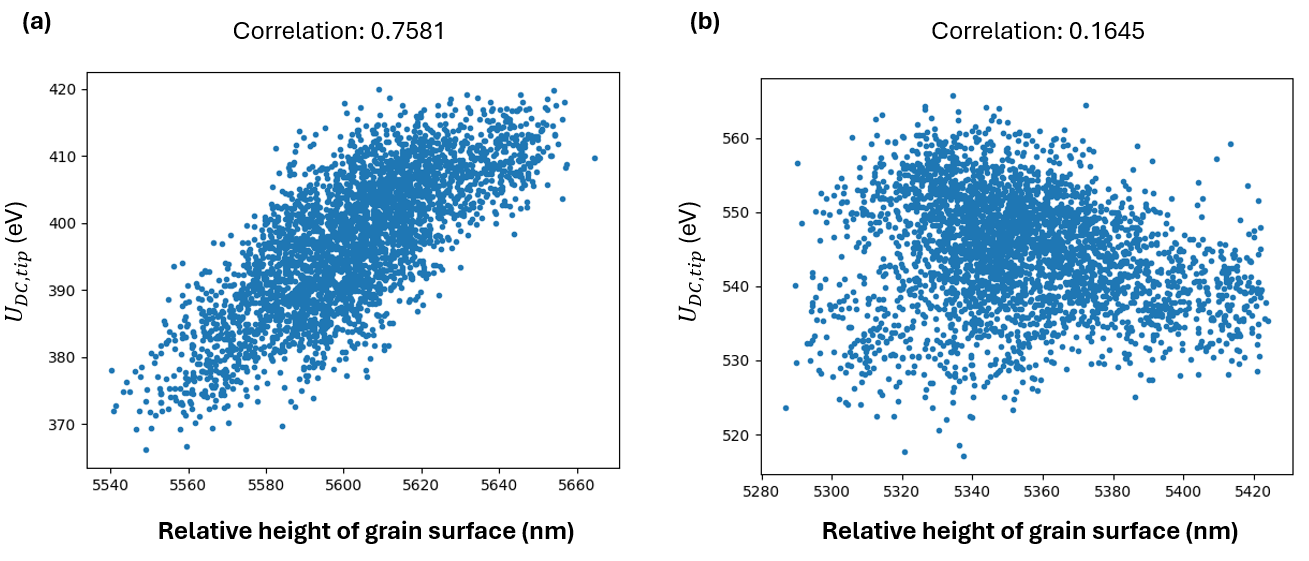


**Figure S11**. Correlation plots between the $U_{DC,tip}$ and the surface topography of the perovskite films (a) **without**, and (b) **with** the PbS layer.


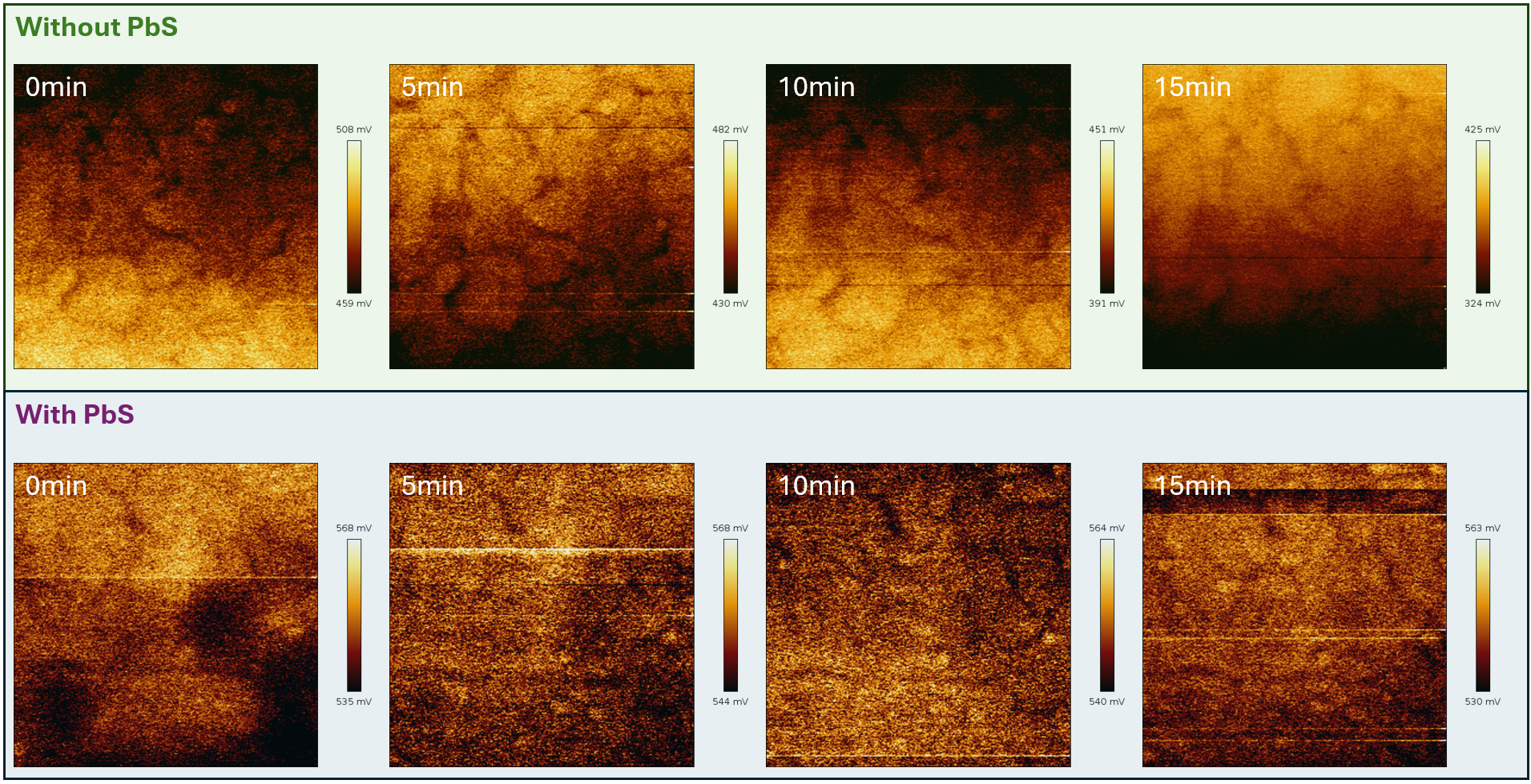


**Figure S12**. KPFM images of the time evolution of the perovskite films **with** and **without** the PbS layer.


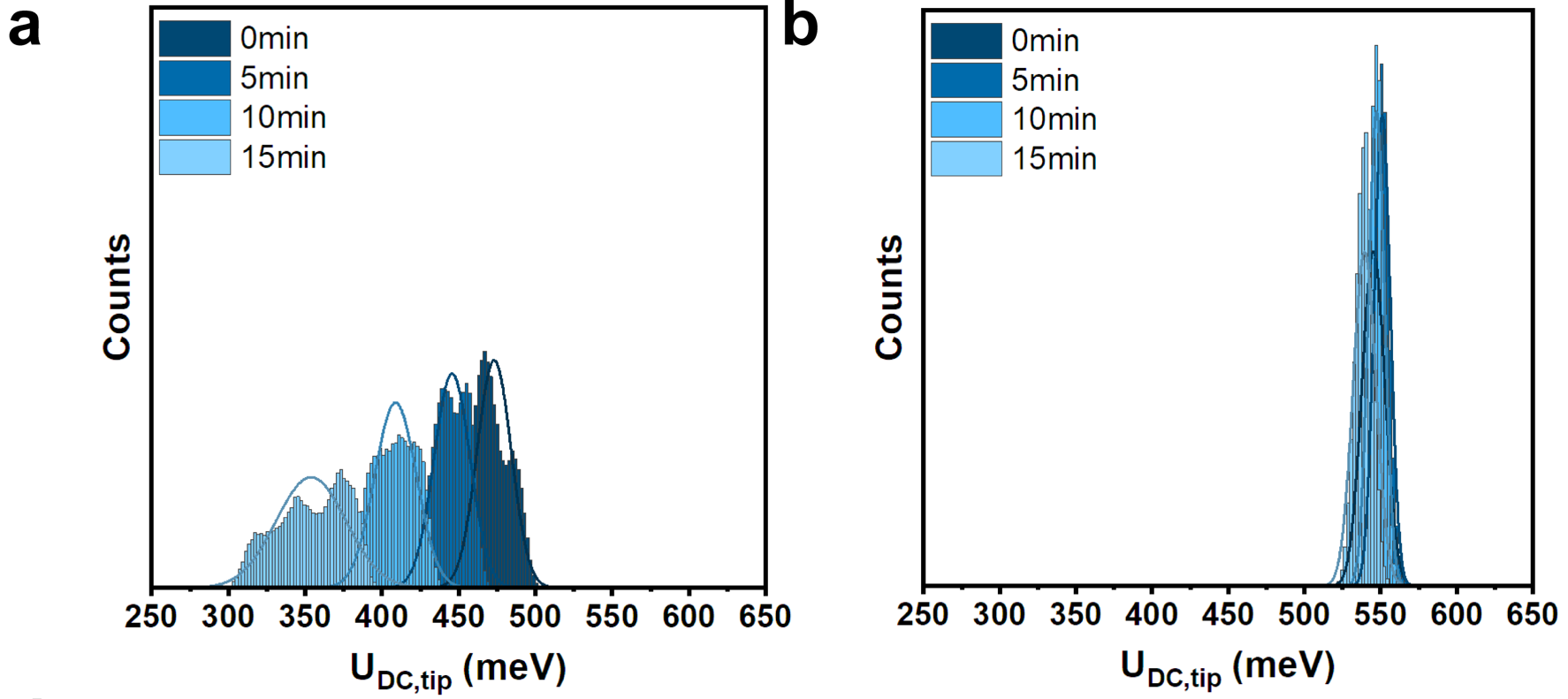


**Figure S13**. Time evolution of the statistical distributions of $U_{DC,tip}$ of KPFM images of the film (a) **without** and (b) **with** the PbS layer over an area of $2\times2\mu m^{2}$.


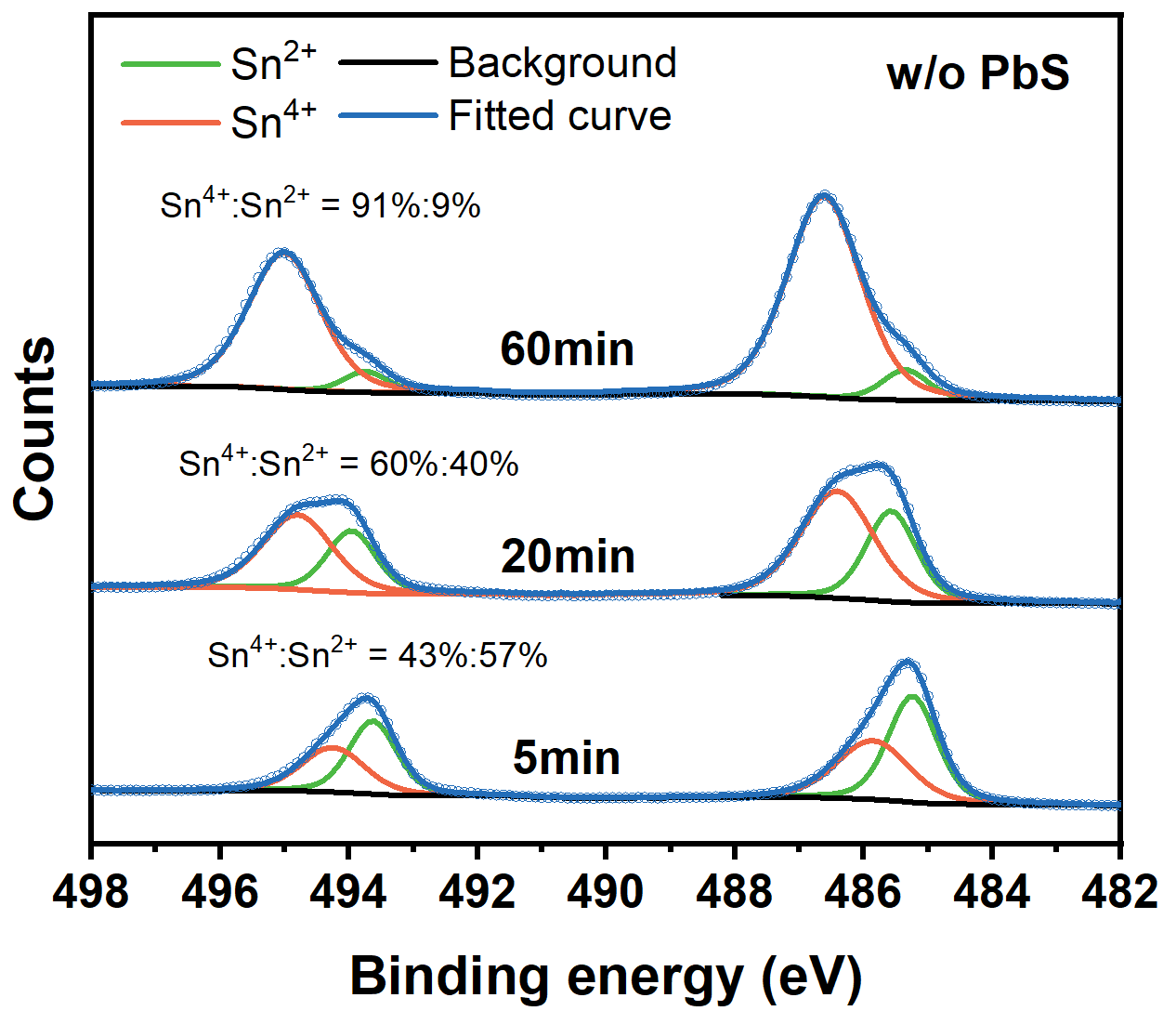

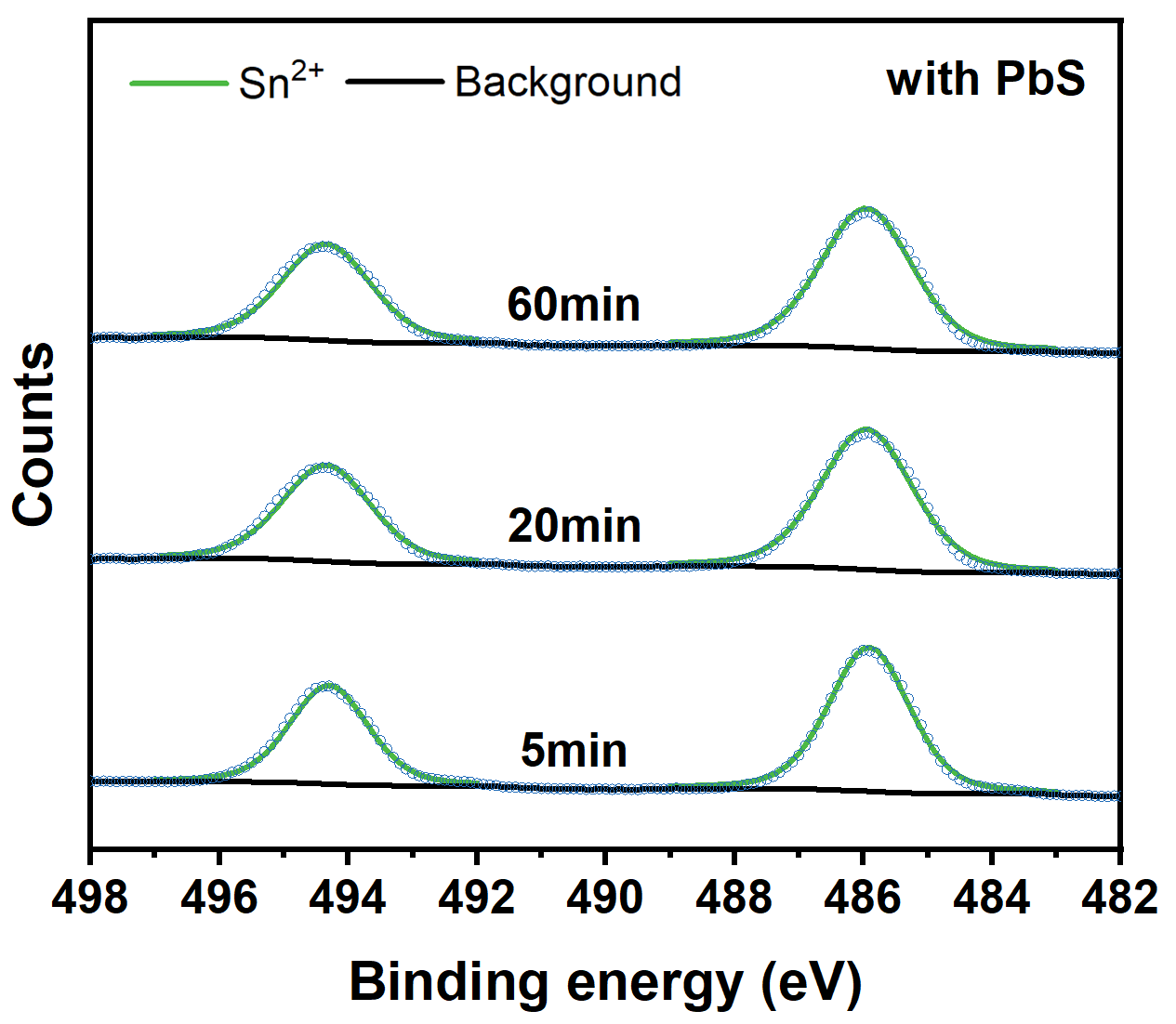


**Figure S14**. Time evolution of the XPS spectra of the aging of the perovskite films (a) **without** and (b) **with** the PbS layer in ambient environment.


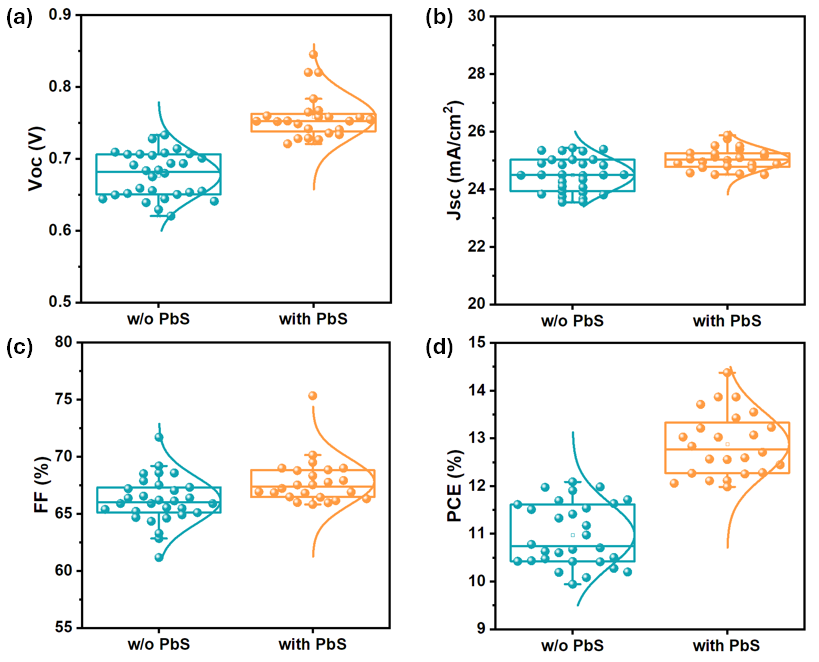


**Figure S15.** Comparison of (a) the open-circuit voltage, (b) the short-circuit current, (c) the fill factor, and (d) the power conversion efficiency of the PSC devices fabricated from the perovskite film **without** and **with** the PbS layer.


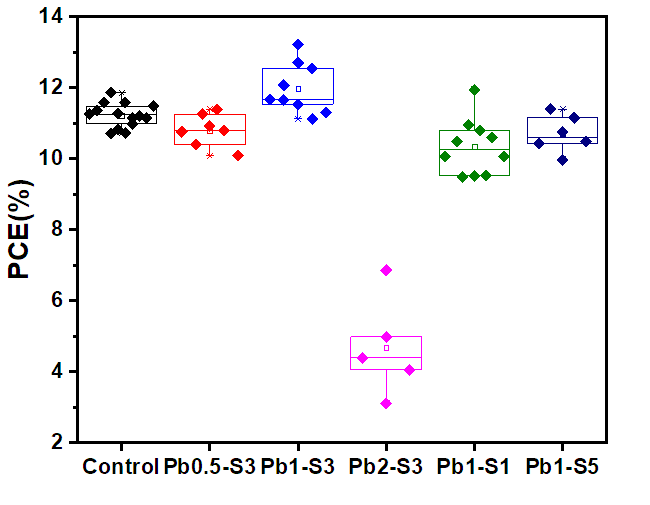


**Figure S16**. Device performance of different precursor concentration combinations. Pb_x_-S_y_ corresponds to a concentration of x mg/mL Pb(PyA)_2_ solution and y mg/mL (TMS)_2_S solution.


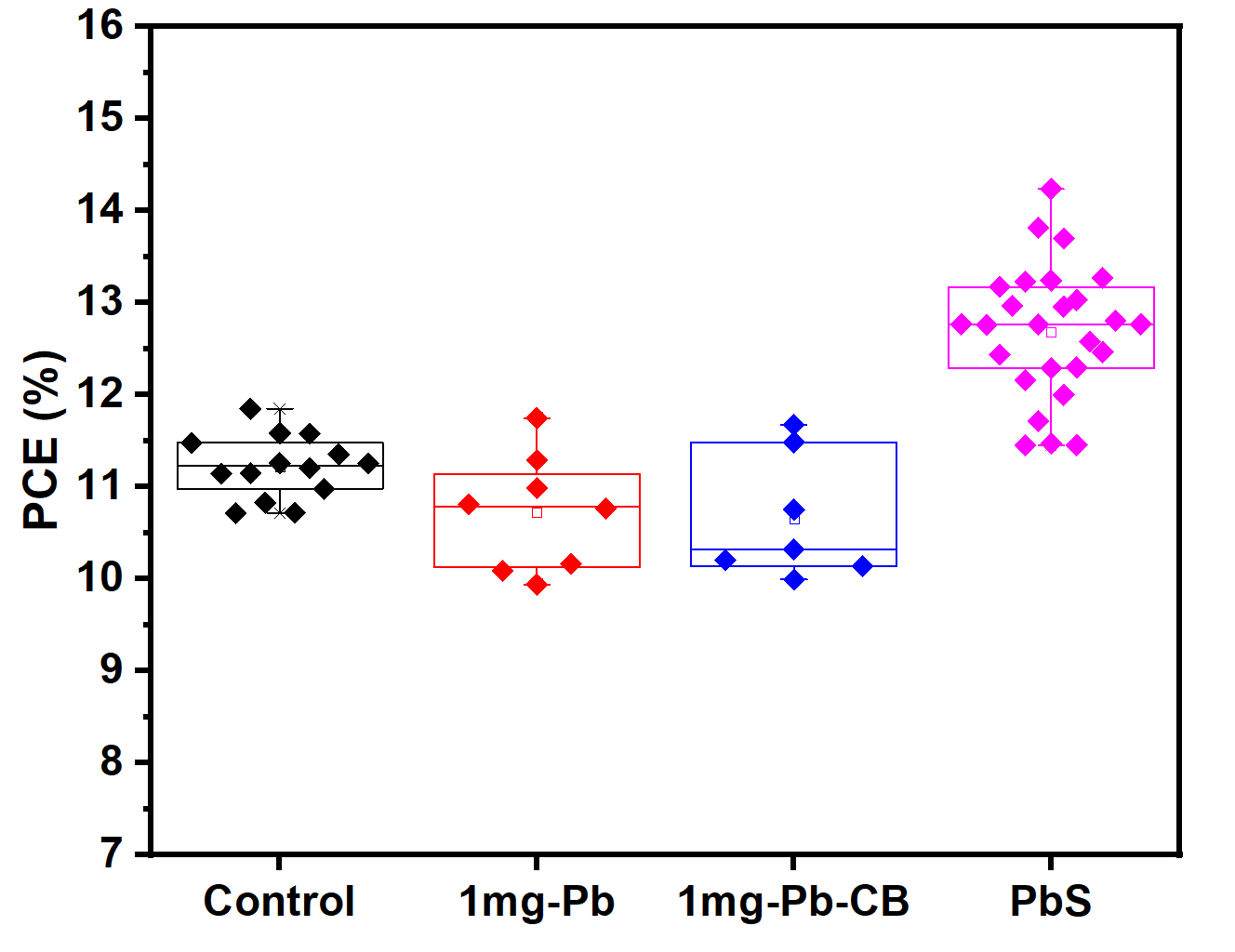


| Conditions | First-step treatment | Second-step treatment |
| --- | --- | --- |
| Control | - | - |
| 1mg-Pb | 1mg/mL Pb(PyA)_2_ | - |
| 1mg-Pb-CB | 1mg/mL Pb(PyA)_2_ | Pure CB |
| PbS | 1mg/mL Pb(PyA)_2_ | 3mg/mL (TMS)_2_S |

**Figure S17**. Device performance of different surface treatments, where the conditions can refer to the table.


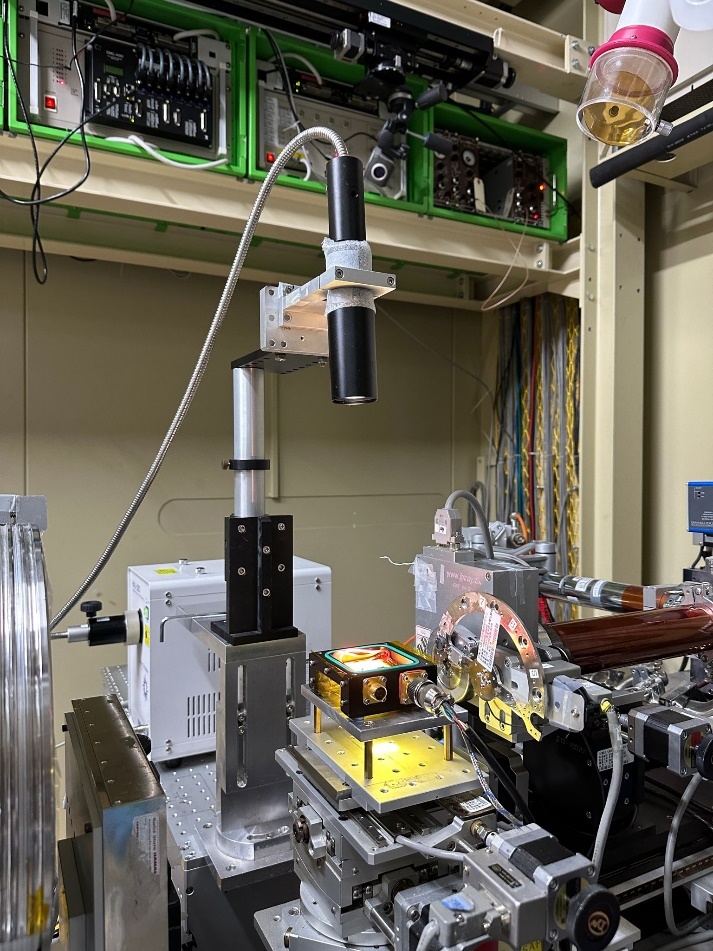

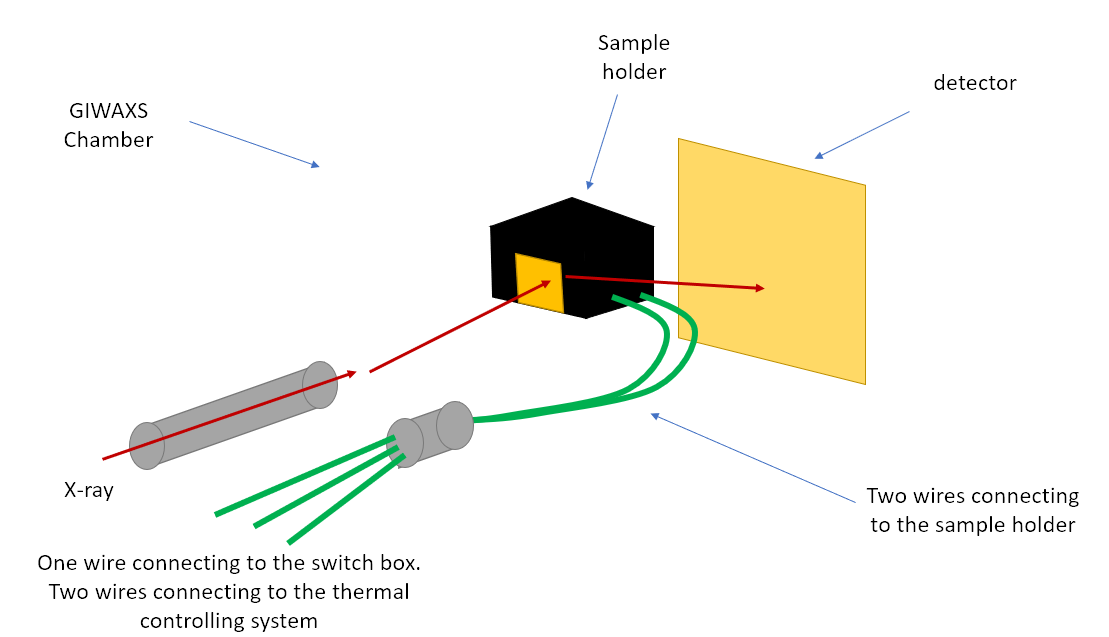


**Figure S18**. Operando GIWAXS measurement setup for the perovskite solar cell device.


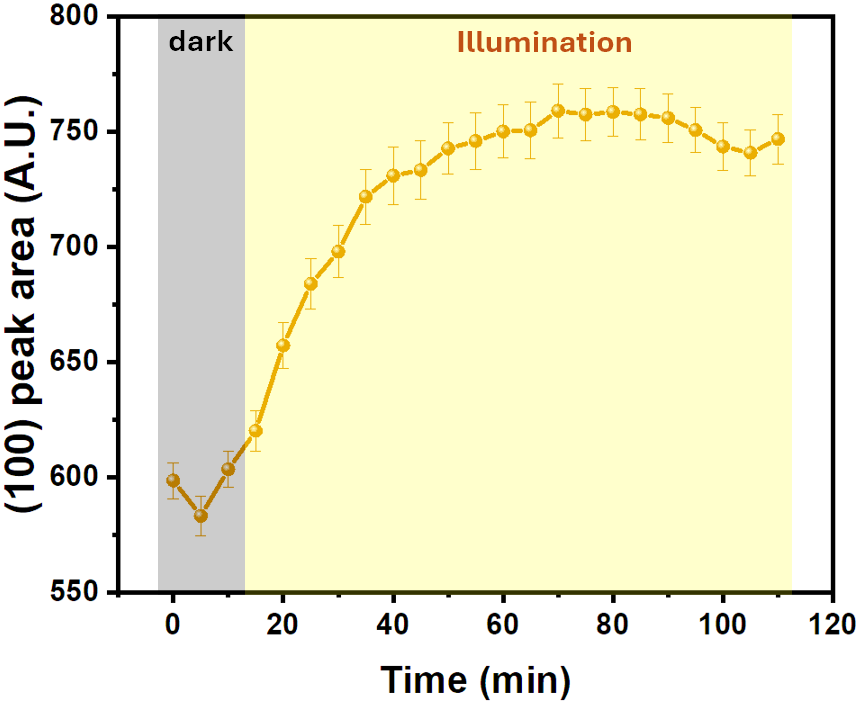


**Figure S19**. *in-situ* GIWAXS measurements under constant illumination in a vacuum condition.


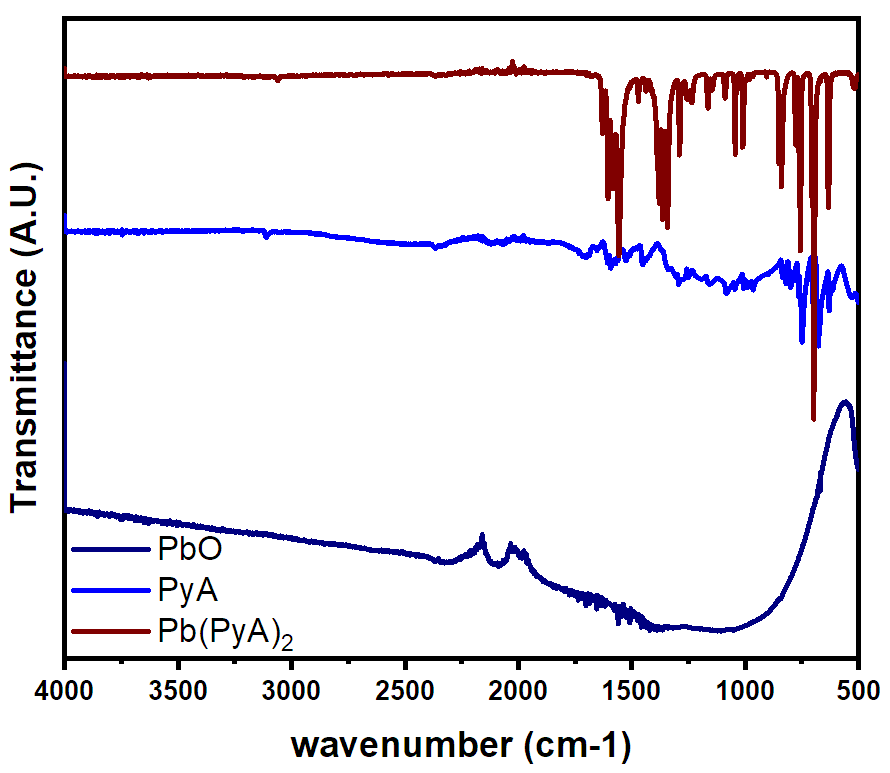


**Figure S20**. Fourier-transform Infrared (FTIR) spectra of PbO, PyA and Pb(PyA)_2_ powder.


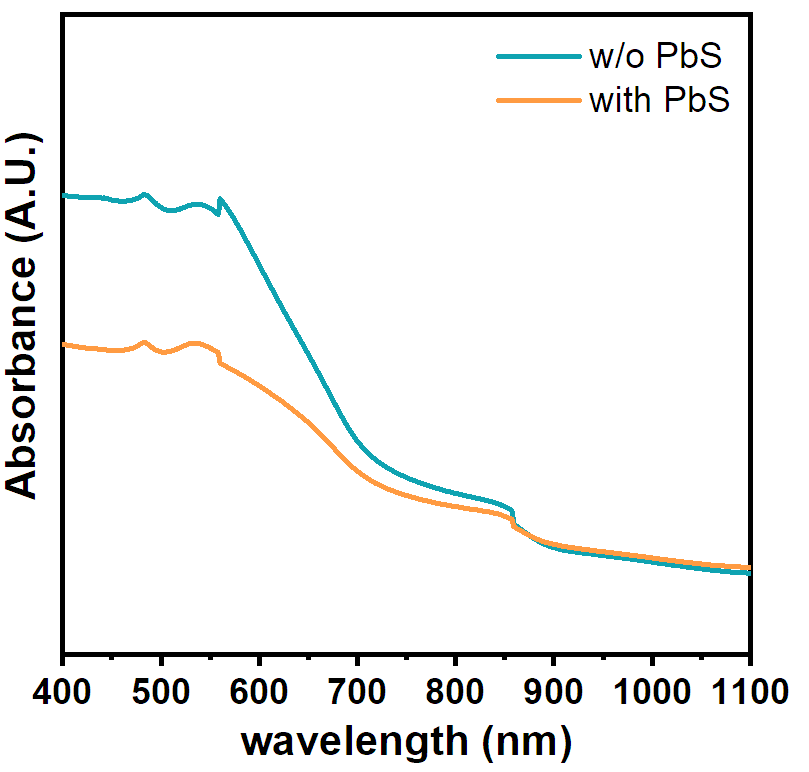


**Figure S21**. UV-Vis spectra of the perovskite film **with** and **without** the PbS layer.

**Supplementary Note 1**. The lead content shown in EDX analysis is directly read out from the measurement software. The lead content deduced from XPS analysis is based on the ratio of area the lead peaks and the tin peaks corrected by the photoionization cross section of the corresponding ions. Similarly, the lead content deduced from ToF-SIMS analysis is based on the counts of the lead and tin signals corrected by their relative ion yield.

**Supplementary Note 2**. Regarding the stability of the PSC devices, moisture plays a critical role together with oxygen. Bare perovskite film exposing ambient air, R.H. = 60%, degraded rapidly in several hours. In contrast, a similar storage in a dry box with R.H. = 25%, the perovskite films retained the black phase after one week. The contact angle measurement shown in **Figure S15** indicates that the water resistance with the iso-BAI treatment is limited. This probably restrict the stability improvement.
